# Diagnostic utility of N-terminal TMPP labels for unambiguous identification of clipped sites in therapeutic proteins

**DOI:** 10.1038/s41598-023-45446-z

**Published:** 2023-10-30

**Authors:** Harsha P. Gunawardena, Meth M. Jayatilake, Jeffery D. Brelsford, Hirsh Nanda

**Affiliations:** 1 Janssen Research and Development LLC, The Janssen Pharmaceutical Companies of Johnson & Johnson, Spring House, PA USA; 2 Department of Oncology, Lombardi Comprehensive Cancer Center, Georgetown University Medical Center, Washington DC, USA

**Keywords:** Mass spectrometry, Structure determination, Chromatography, Mass spectrometry, Antibody therapy

## Abstract

Protein therapeutics are susceptible to clipping via enzymatic and nonenzymatic mechanisms that create neo-*N*-termini. Typically, neo-*N*-termini are identified by chemical derivatization of the *N*-terminal amine with (N-Succinimidyloxycarbonylmethyl)tris(2,4,6-trimethoxyphenyl)phosphonium bromide (TMPP) followed by proteolysis and mass spectrometric analysis. Detection of the TMPP-labeled peptide is achieved by mapping the peptide sequence to the product ion spectrum derived from collisional activation. The site-specific localization of the TMPP tag enables unambiguous determination of the true N-terminus or neo-N-termini. In addition to backbone product ions, TMPP reporter ions at *m/z* 573, formed via collision-induced dissociation, can be diagnostic for the presence of a processed *N*-termini. However, reporter ions generated by collision-induced dissociation may be uninformative because of their low abundance. We demonstrate a novel high-throughput LC–MS method for the facile generation of the TMPP reporter ion at *m/z* 533 and, in some instances *m/z* 590, upon electron transfer dissociation. We further demonstrate the diagnostic utility of TMPP labeled peptides derived from a total cell lysate shows high degree of specificity towards selective N-terminal labeling over labeling of lysine and tyrosine and highly-diagnostic Receiver Operating Characteristic’s (ROC) of TMPP reporter ions of *m/z* 533 and *m/z* 590. The abundant generation of these reporters enables subsequent MS/MS by intensity and ***m/z***-dependent triggering of complementary ion activation modes such as collision-induced dissociation, high-energy collision dissociation, or ultraviolet photo dissociation for subsequent peptide sequencing.

## Introduction

Advances in protein engineering have enabled novel therapeutic modalities that are unique in structural and conformational diversity^1^. However, protein-based drugs are prone to proteolytic degradation that may impair efficacy and safety. For example, clipping of recombinant proteins is ubiquitous and can occur in culture or during process development. Clipping is often attributed to host cell site-specific or broad specificity proteases^2,3^. Clipping can also occur by nonenzymatic mechanisms depending on the type of side chain, alteration of local flexibility due to secondary, tertiary and quaternary structures, and pre-analytical variables (e.g., pH, temperature, metals and radicals) used to assess developability^4,5^. The amounts of the clipped species and the analytical methods used to monitor purity and integrity are critical quality attributes of the product or process^6,7^.

Mass spectrometry with several different emerging technologies is an indispensable tool for the characterization of therapeutic proteins^8,9^. The complete characterization of a protein therapeutic requires determination of primary amino acid sequence, detection of unprocessed signal peptides, sequence variant analysis, identification and localization of post-translational modifications (PTMs), and process-related modifications^10–15^. Accurate identification and site-specific localization of these attributes can benefit from tandem mass spectrometry (MS/MS) dissociation methods capable of providing complementary information about a polypeptide. Electron transfer dissociation (ETD) has enabled localization of fragile PTMs^16^ within the peptide backbone while collision-based dissociation methods such as collision-induced dissociation (CID) and high-energy collision dissociation (HCD) can dissociate labile bonds of PTMs to determine subunit connectivity of complex polymeric PTMs such as glycans^17^. Ultraviolet photodissociation (UVPD) in combination with multiple dissociation modes has also been shown to complement HCD and ETD and increase overall sequence coverage in top-down protein characterization^18^.

In addition, different dissociation modes produce immonium ions due to side-chain fragmentation. These ions give rise to unique low-mass signature ions that are diagnostic of sequence composition or PTM^19^. The generation of low ***m/z*** diagnostic ions assists in subsequent structural elucidation by triggered ion scan functions on hybrid orbitraps^20,21^. Signature ions that are diagnostic of modifications have been analytically useful. These diagnostic ions can be generated via different tandem MS techniques. For example, HCD has been used to examine lysine glycation^22^, Selective ETD of N-linked glycopeptides has been performed via HCD derived oxonium ions^23,24^, and identification peptides containing lysine modifications has been performed using precursor ion scanning technique with beam-type CID^25,26^.

Shotgun mass spectrometry characterization of protein clipping sites is challenging due to preferential sequencing of the most abundant peptides in a protein digest. Low stoichiometry of a clipped species can prevent detection of peptides having neo-*N*-termini. In addition, in-solution and in-source fragmentation artifacts can cause false-positive identification of neo-*N*-terminal peptides sequenced by mass spectrometry. Investigators have described several analytical strategies to circumvent these limitations^27–29^. Selective *N*-terminus labeling with N-tris(2,4,6-trimethoxyphenyl)phosphonium bromide (TMPP) is well-suited for subsequent mass spectrometric analysis because this labeling reagent increases the hydrophobicity of *N*-terminal peptides, improves their ionization ability, and modifies their fragmentation pattern due to the introduced positive charge^30^. TMPP-labeled peptide dissociation has been examined by post-source decay and CID in which diagnostic ions were observed. Gunawardena et al. demonstrated that ETD of TMAB fixed charged derivatized peptides gave rise to backbone product ion abundances different from their protonated counterpart^31,32^. Furthermore, Zimnicka et al. showed that tunable charge-tags enabled N-terminal sequence ions to resolve ambiguities in fragment ion assignment^33^. However, for clipping assessment, the diagnostic utility of generating charge-loss product ions of a fixed charged derivatizing agent has not been demonstrated.

Here we demonstrate the utility of TMPP-derived reporter ions to identify clipped peptides via ETD-MS2 and diagnostic ion-triggered MS2 events to autonomously filter clipped peptides. We demonstrate our approach for efficient generation of reporter ions of TMPP-labeled standard peptides that represent both *N*-terminal clipped species and undesirable TMPP labeling of lysine and tyrosine residues. In addition, we used a large pool of TMPP-labeled peptides with varying sequences, lengths, and charge states to demonstrate the diagnostic practicality of reporter ions generated via ETD-MS2 compared with HCD-MS2. We used this approach to examine the sequential clipping of the GLP1 peptide from the commercially available GLP1 agonist Dulaglutide which we treated with Cathepsin D. In comparing the utility of TMPP reporter ions for complementary dissociation modes, ETD, HCD, CID and UVPD, we found that the facile charge loss peak at *m/z* 533 of TMPP + ion generated via ETD was the most diagnostic for a TMPP-labeled peptide having a neo-*N*-terminus. Lastly, we show that TMPP-labeled peptides are rapidly (< 20 min) and efficiently separated from unlabeled peptides in complex samples, which enhances the retention time predictability of the TMPP-labeled peptides which improves the specificity and reduces false-positive identification of clipped peptides.

## Materials and methods

### Chemicals and reagents

(N-Succinimidyloxycarbonyl)tris(2,4,6-trimethoxyphenyl)phosphonium bromide (TMPP), *4-*Morpholineethanesulfonic acid monohydrate (MES), N-(2-Hydroxyethyl)piperazine-N′-(2-ethanesulfonic acid) (HEPES), Trimethyl ammonium bicarbonate (TMAB), and sodium phosphate dibasic (Na_2_HPO_4_), sodium phosphate monobasic (NaH_2_PO_4_), Dimethylformamide (DMF), Cathepsin D from bovine spleen, 1,4 dithiothreitol (DTT), Iodoacetamide (IAA) and NIST-IgG1-K1 monoclonal antibody were all purchased from Sigma (St Louis, MO). Peptide standards from NIST monoclonal antibody was synthesized to 99.99% purity by Biomatik Corporation (Ontario, Canada). Human K562 predigest cell extract and sequencing grade Trypsin and endoproteinase Lys-C were purchased from Promega (Madison, WI). GLP1-Fc fusion protein was purchased from Myoderm (Norristown, PA). Optima LC–MS grade acetonitrile, water, formic acid, hydroxylamine, and Gibco PBS buffer solution were all purchased from Thermofisher Scientific (Waltham, MA).

### N-terminal labeling and protein digestion

Synthetic peptides, peptides from K562 predigest, NIST monoclonal antibody, and GLP1-Fc fusion protein were derivatized with TMPP. Derivatization was performed in the following buffers: 100 mM each of MES pH 6, HEPES pH 7, and sodium phosphate pH 8. A fresh 100 mM TMPP solution was prepared by dissolving 100 mg in 1.3 mL of DMF. TMPP labeling was performed by adapting a published derivatization protocol^27^. In brief, 10 µL TMPP solution was added to 50 µg of peptides and proteins and mixed briefly followed by adding 40 µL of the buffer and incubated for 1 h. The reaction was quenched with 1 µL of hydroxylamine and lyophilized to dryness. The dried peptides were reconstituted with 0.1% formic acid–water for MS, and the dried proteins were reconstituted with TMAB for trypsinization. Proteins were digested as described^34^. Briefly, proteins were reduced with DTT and alkylated with iodoacetamide. The proteins were then subjected to proteolysis with endoproteinase Lys-C for 1 h at 37 °C followed by fourfold dilution in 25 mM TMAB, pH 8.0, 1 mM CaCl_2_ and digested with trypsin for 4 h at 37 °C. Digestion was stopped by the addition of formic acid to 0.1%. The peptide solutions were desalted on Sep-Pak Light C18 cartridges (Waters, Milford, MA) and collected for mass spectrometry.

### Instrumentation

All analysis was performed with an Agilent 1200 HPLC (Agilent Technologies, Santa Clara, CA) coupled to an Orbitrap Lumos (Thermo Scientific, San Jose, CA) Tribrid mass spectrometer equipped with an electrospray ion source using tune application software 2.1.1565.18 and Xcalibur 4.0.27.13.

### LC–MS/MS analysis

All samples subjected to LC–MS/MS analysis were separated on an Agilent Infinity 1290 UHPLC (Agilent Technologies, Santa Clara, CA) using an AdvanceBio Peptide Map Micro Bore Rapid Resolution Column (1 × 150 mm, 2.7 µm) column at 65^o^ C. The column was elute with a 20-min rapid LC gradient program using water with 0.1% formic acid as mobile phase A and acetonitrile with 0.1% formic acid as mobile phase B was employed: 0 min, 2% B; 10 min, 30% B; 10.5 min, 2% B; 11.5 min, 85% B; 12 min, 2% B; 13 min, 85% B; 13.5 min, 2% B; followed by wash step from 14-18 min, 85% B; and a subsequent re-equilibration for 2 min at 2% B. The 120 min LC gradient method utilized water with 0.1% formic acid as mobile phase A and acetonitrile as mobile phase B with 0.1% formic acid was employed: 0 min, 2% B; 60 min, 30% B; 60.5 min, 2% B; 61.5 min, 85% B; 62 min, 2% B; 63 min, 85% B; 63.5 min, 2% B; followed by wash step from 64.5 to 80 min, 85% B; and a subsequent re-equilibration for 20 min at 2% B. The flow rate in all gradients was set to 0.2 mL/min and the injection volume was 2 µL. The mass spectrometer was operated in positive ionization mode with a data dependent MS^2^ ETD, CID, HCD, UVPD methods. The interface conditions were as follows: emitter voltage, -2600 V; vaporizer temperature, 325^o^ C; ion transfer tube, 325 °C; sheath gas, 55 (arb); aux gas, 10 (arb); and sweep gas, 1 (arb).

### Method settings

The internal mass spectrometer settings used for MS scans, unless stated otherwise, were the following: RF lens, 60%; AGC target, 4e5; maximum injection time, 50 ms; and 1 µscan in profile mode at 50 K resolution on the Orbitrap mass analyzer. The method then sequentially included a series of filters prior to any HCD MS^2^ events. A monoisotopic peak selection filter was included and set as peptide for all methods. An intensity filter of 1e5 was used for all methods unless stated otherwise. An optional charge state filter was included for some methods to select precursor charge states 2–6. An optional dynamic exclusion (DE) filter was included for some methods with either a 12 s or 3 s exclusion window and which had the following common parameters: exclude n = 1 times; + /− 3 ppm; exclude isotopes; and single charge state per precursor. Apex detection was included for one method and was set to expected peak width, 6 s and desired apex window 30%. There were five ddMS^2^ OT-ETD scans with the following settings unless stated otherwise: quadrupole isolation, 2 m*/z* isolation window; reaction time of 50 ms detector type, Orbitrap, auto *m/z* normal scan range, 15 K resolution, 100 m/z first mass; AGC Target, 2e5, inject ions for all available parallelizable time, 50 ms maximum injection time; 1 µscan, profile. A targeted mass trigger followed ddMS^2^ IT-CID and included ions 533.193, 590.214; + /− 5 ppm error tolerance; with the detection of either 2 or 1 ions from the list as explicitly stated; only ions within the top 10 most intense for all mass triggers. Subsequent ddMS^2^ OT-CID conditions were as follows unless stated otherwise: MS^n^ Level, 2; quadrupole isolation, 1.6 m*/z* isolation window; CID collision energy, 30; activation Q, 0.25; detector type, Orbitrap, auto *m/z* normal scan range, 15 K resolution; AGC Target, 5e4, inject ions for all available parallelizable time, 22 ms maximum injection time; 1 µscan, profile. The number of dependent scans between ddMS^2^ OT-ETD and ddMS^2^ IT-CID was set to 1. There were five ddMS^2^ OT-HCD scans with the following settings unless stated otherwise: quadrupole isolation, 1.6 m*/z* isolation window; HCD collision energy, 40%, stepped 5%; detector type, Orbitrap, auto *m/z* normal scan range, 15 K resolution, 100 m/z first mass; AGC Target, 5e4, inject ions for all available parallelizable time, 35 ms maximum injection time; 1 µscan, profile.

### Data analysis

Data analysis was performed with Xcalibur visualization software from Thermo Scientific (San Jose, CA), Byos 3.9 chromatography and mass spectrometry data analysis software from Protein Metrics (Cupertino, CA), and R 3.6 and 4.3 statistical programming software (Vienna, Austria).

## Results and discussion

### Detection of diagnostic ions by TMPP labeling of N-termini and electron transfer dissociation

**Figure 1:**
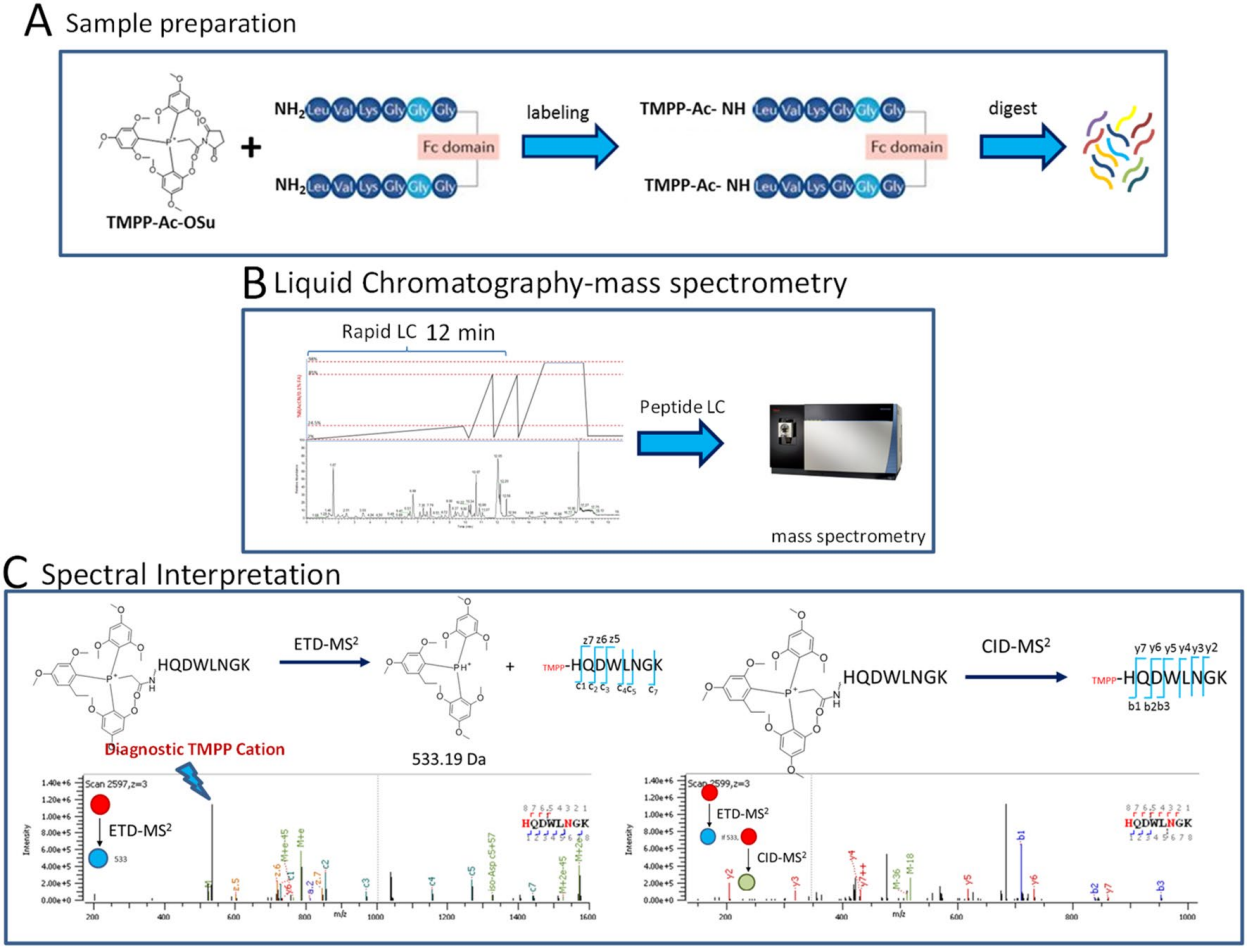
Clip site identification strategy (**A**). TMPP labeling of N-termini or Neo N-termini of clip-sites and proteolytic digestion (**B**)*.* Rapid chromatographic separation of tryptic peptides and mass spectrometry (**C**). Interpretation of TMPP diagnostic ion-triggered mass spectra where ETD-MS2 generates diagnostic reporter ions TMPP + of clipped peptides and TMPP + diagnostic ions trigger a CID-MS2.

We first examined the diagnostic utility of the TMPP reporter ions by labeling the NIST antibody with TMPP followed by tryptic peptide mapping by data-dependent ETD-MS/MS. Figure 1 shows a workflow that consists of three steps that facilitate the facile identification of clip sites; *A.* TMPP labeling of N-termini or Neo N-termini of clip-sites and proteolytic digestion, *B.* Rapid chromatographic separation of tryptic peptides and *C.* Interpretation of TMPP diagnostic ion-triggered mass spectra. The NIST antibody has authentic *N*-termini from the light and heavy chains. One of the two surrogate peptides corresponded to the light chain *N*-terminus that has a free primary amine, whereas the *N*-terminus of the heavy chain has a secondary amine due to cyclization of glutamine to pyroglutamic acid. Any neo-*N*-termini on the NIST antibody was a potential degradation or clipping product that arose during storage.

**Figure 2:**
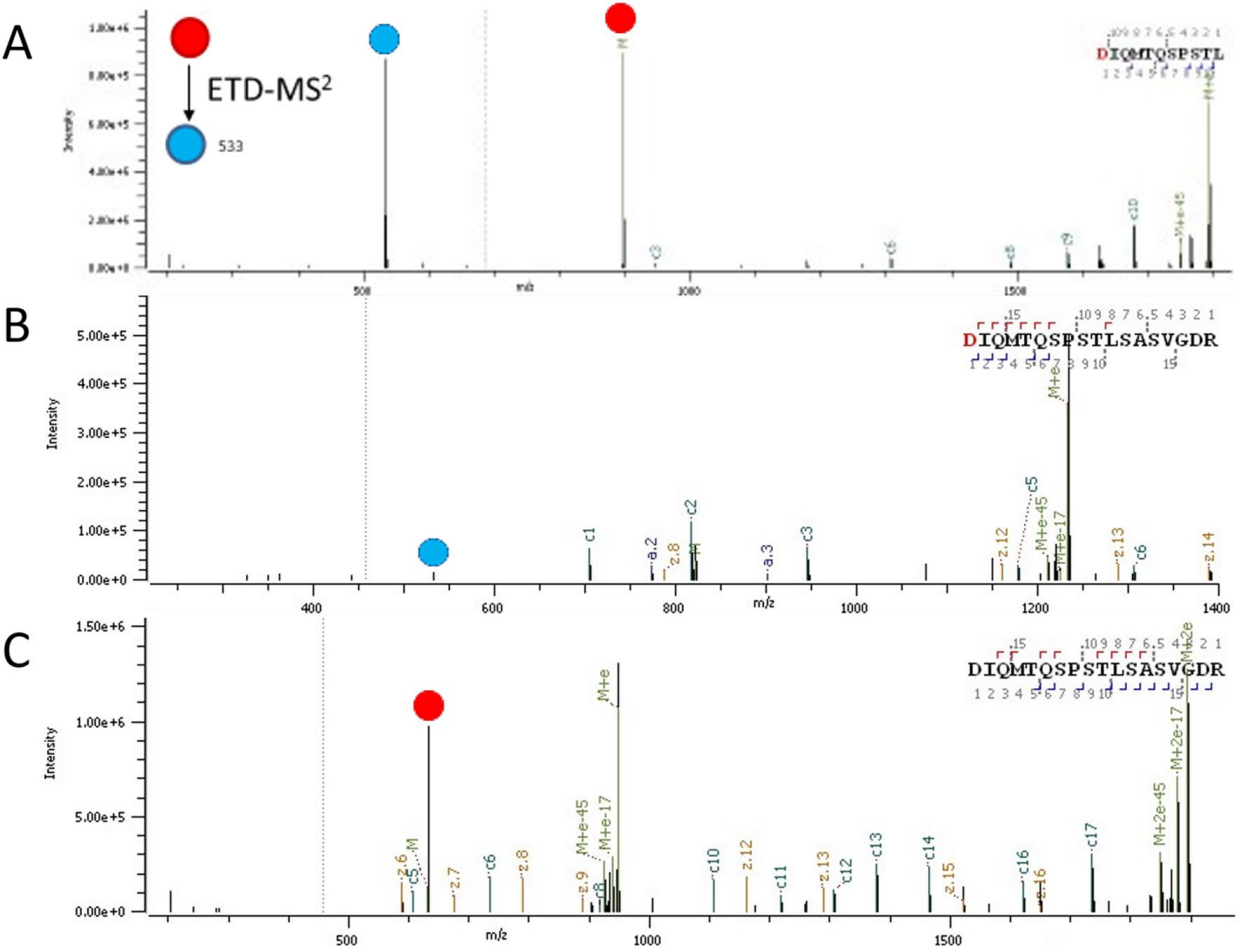
Product ion spectrum of *N*-terminal sequences of the light chain of NIST antibody where precursor and diagnostic ions are denoted by red and blue circles respectively (**A**) ETD-MS2 of TMPP labeled doubly charged peptide fragment (**B**) ETD-MS2 of TMPP labeled triply charged tryptic peptide (**C**) ETD-MS2 of unlabeled triply charged tryptic peptide.

Figure 2a shows the ETD product ion spectrum of the peptide corresponding to the light chain *N*-terminal sequence of the NIST antibody. The mass spectrum consists predominantly of the diagnostic TMPP + reporter ion (*m/z* 533) and a *c.*-type backbone product ion that consists of the *N*-terminal TMPP tag. Note that this non tryptic peptide sequence was generated because of in-source fragmentation of a larger tryptic peptide **(**Fig. 2b**)** that produced the reporter ion to a much lower degree during ETD, presumably due to charge sequestration on the *C*-terminal arginine residue^32^ and the corresponding unlabeled peptide (Fig. 2c) shows no diagnostic ions. Nevertheless, the ETD approach produced a dominant reporter ion peak, it localized the TMPP tag on the *N*-terminus using high-sequence coverage, and reporter ion triggering of a complementary activation event confirmed the presence of a true light chain *N*-terminus. In addition, by filtering ETD-MS/MS spectra that had *m/z* 533 and *m/z* 590 diagnostic reporter ions in the entire data set, we confirmed the absence of additional low-level clipped species resulting from degradation of the NIST antibody. Our triggered CID approach made the data filtering amenable to manual inspection due to the low occurrence of triggered MS2 scans that confirm the presence of reporter ions generated from ETD-MS/MS events for the entire data set. Triggered scans eliminate the need for in-slico approaches or manual inspection of ETD-MS/MS scans that have reporter ion peaks.

### Rapid separation of TMPP labeled peptides and retention time predictability

**Figure 3:**
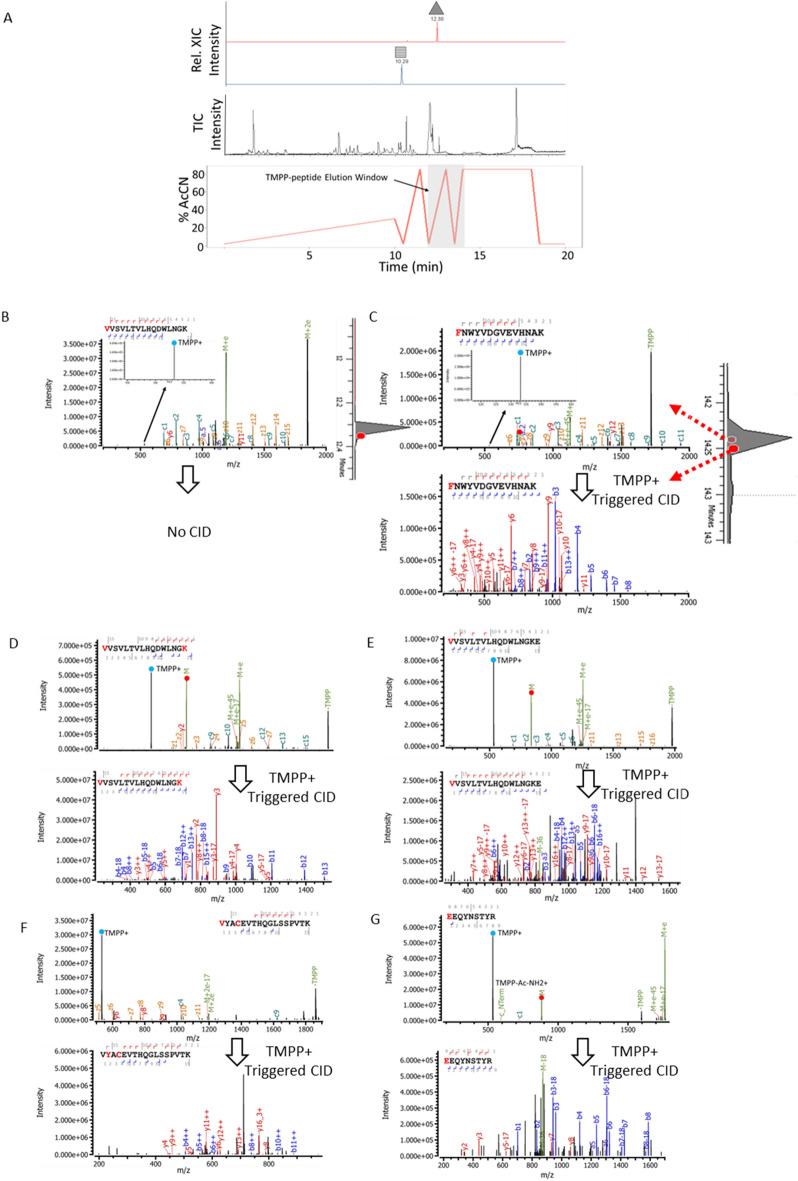
Rapid separation and ETD-MS2 and TMPP + diagnostic ion triggered CID-MS2 of peptides derived from NIST mAb after TMPP labeling (**A**) The total Ion Chromatogram (TIC) of peptides with TMPP derivatized peptides eluting 12–14 min retention time window. Inset shows Extracted Ion Chromatograms (XICs) of labeled () and unlabeled () NIST *N*-terminal peptide (**B**). ETD-MS2 of N-terminus TMPP labeled peptide VVSLTVLHQDWLNGK (3 +) with Inset shows lower intensity TMPP + diagnostic ions and absence of triggered CID-MS2. Inset chromatogram shows MS2-ETD. (**C**) ETD-MS2 of N-terminus TMPP labeled FNWYVDGVEVHNAK (3 +) with inset shows lower intensity TMPP + diagnostic ions that triggered CID-MS2. Inset chromatogram shows MS2-ETD and MS2-CID. (**D**) ETD-MS2 of TMPP labeled at N-terminus and Lysine of VVSLTVLHQDWLNGK (3 +) and TMPP + triggered CID-MS2 (**E**) ETD-MS2 of TMPP labeled Lysine and methylated N-terminus of VVSLTVLHQDWLNGKE (3 +) and TMPP + triggered CID-MS2 (**F**) ETD-MS2 spectrum of single TMPP labeled peptide VYACEVTHQGLSSPVTK and corresponding TMPP + triggered CID-MS2. (G) ETD-MS2 spectrum of a single TMPP labeled peptide EPQVYTLPPSR and corresponding TMPP + triggered CID-MS2.

We next evaluated rapid separation conditions for high-throughput detection of TMPP-labeled peptides. We achieved complete reverse-phase separation of peptides, clean-up, and re-equilibration within 20 min. Figure 3 shows the reverse-phase chromatographic buffer gradient and the corresponding total ion chromatogram of the NIST digest after TMPP labeling. Importantly, most unlabeled peptides eluted at 2–30% organic solvent in a 10-min shallow gradient. The NIST antibody *N*-termini surrogate peptides of the light chain eluted at 12 min. TMPP labeling increases the hydrophobicity of *N*-terminal peptides, and it is conceivable that TMPP-labeled peptides were observed mostly during the two sharp gradients with rapid ramps: 2–85% of organic solvent in 1 min. The ability to separate and improve the retention time predictability of the TMPP-labeled peptides improves the specificity and reduces false-positive peptide identification. Notably, we also observed the corresponding unlabeled NIST mAb N-terminal peptide counterpart at ~ 10 min. Complex samples having multiple clipped species will consists of TMPP-labeled and unlabeled peptide counterparts (due to < 100% labeling efficiency). Compared with a labeled peptide, an unlabeled peptide elutes earlier and is usually lower in intensity, sometimes below the LOD.

Next, we derivatized and combined 15 synthetic peptide standards from the NIST antibody sequence to assess the likelihood of observing unlabeled peptides and to evaluate the retention time predictability of TMPP-labeled peptides. We recorded the retention times and intensities of unlabeled and TMPP-labeled peptides in the 20-min HPLC gradient injection. The reaction efficiency for TMPP labeling was assessed from a peak area ratio of the TMPP-labeled peptide normalized to the total intensity for that peptide. The 15 TMPP peptides eluted between 12 and 14 min (Fig. 3a) with labeling efficiency from 8 to 100%; eight of the 15 peptides reacted with 100% efficiency (Table 1). All TMPP-labeled peptides were identified by MS/MS. The unlabeled peptide counterparts eluted with a much wider retention time window of 4–12 min. Due to the high reaction efficiency, we identified only 7 of 15 unlabeled peptides by MS/MS. The remaining 8 unlabeled peptides were detected at MS1, yet they did not trigger MS2-ETD due low LOD. In general, peptide length of ≤ 5 residues most often completely derivatized. Longer peptides and N-terminal pyroE generally had the lowest N-terminal %TMPP conversion. It was also notable that TMPP had a propensity to label Y residue in peptides that had length of > 5. Also, the preference of Y over N-terminus increased as the overall peptide length increased. Peptide labeling enables improved identification and retention time predictability. The LC run times of the overall gradient can be shortened for rapid identification of clip sites for low complexity samples or extended for more complex mixtures.

**Table 1:** ETD efficiency of TMPP labeled NIST synthetic peptides. Note: reporter ion ETD efficiencies are from Eq. 1, and overall ETD efficiency are from Eq. 4 and Eq. 5  (Supplement 1).

Sequences	Sequence Length	%TMPP derivatization	Y Counts	K Counts	Obs m/z	Charges	% ETD (TMPP +) Eq 1	% ETD (TMPP-Ac-NH2+) Eq 1	% Overall ETD Efficiencies Eq 4	% Overall Efficiencies No Reporters Eq 5
tmpp-HK	2	100	0	1	286.1224	3	26.86	4.4	93.5	62.2
tmpp-CK	2	100	0	1	411.1514	2	46.78	0.0	100.0	45.6
tmpp-HK(tmpp)	2	100	0	1	428.6803	2	36.98	1.1	73.4	33.1
tmpp-EAK	3	100	0	1	460.1910	2	39.62	11.4	93.8	39.8
tmpp-EYK	3	100	1	1	506.2039	2	39.57	17.6	94.0	34.1
tmpp-TKPR	4	100	0	1	515.2209	2	48.72	0.0	84.1	31.6
tmpp-SFNR	4	100	0	0	537.2517	2	40.07	0.5	80.4	35.8
tmpp-VQWK	4	100	0	1	548.2256	2	50.55	1.2	92.4	36.5
VY(tmpp)ACEVTHQGLSSPVTK	4	97.7 (Y2)	0	1	566.7540	2	49.87	1.8	92.6	34.9
tmpp-ADYEK, ADY(tmpp)K	17	66(N-term), 6 (Y6)	1	1	598.5279	4	35.94	0.0	100.0	64.1
tmpp-GQPR	5	100	0	1	599.2361	2	43.48	3.0	92.3	42.3
tmpp-DTLMISR	7	50.4	0	0	704.3136	2	46.28	0.9	72.3	21.2
tmpp-FNWYVDGVEVHNAK	14	52.3	1	1	750.6667	3	0.28	0.0	100.0	99.7
tmpp-VVSVLTVLHQDWLNGK	16	58.9	0	1	794.0677	3	0.27	0.0	89.0	88.7
tmpp-EEQYNSTYR	9	22, 4(PyroE)	2	0	881.3501	2	31.90	0.4	66.7	31.5
tmpp-EPQVYTLPPSR	11	8.2, 14.6(PyroE)	1	0	929.9333	2	3.88	0.0	66.6	62.6

### Diagnostic ions of TMPP-labeled synthetic peptide standards

We derivatized several NIST mAb synthetic peptide standards with TMPP and subjected them to LC–MS using ETD-MS2 and diagnostic reporter ion triggered-MS2-CID events. The synthetic peptides had different lengths, amino acid compositions, and sites of TMPP labeling. We evaluated the propensity of the peptides to generate diagnostic TMPP reporter ions (*m/z* 533, and *m/z* 590) in the ETD spectra. We derived several efficiency estimates of the ETD spectra of NIST peptides (Fig. S1) for TMPP and TMPP-Ac + reporter ions for TMPP derivatized peptides based on % efficiency calculations reported for fragmentation of peptide backbone bonds^35^. Table 1 shows that most peptides triggered a subsequent MS2-CID scan upon detection of TMPP reporter ions (Fig. S2). The triggered scans were dependent on the intensity of the reporter ion TMPP + *m/z* 533. From synthetic NIST peptides, we observed that TMPP + efficiencies were significantly greater than the TMPP-Ac + efficiencies due to reporter ion abundance differences. Interestingly, the reporter ion intensity contributed significantly to the overall ETD efficiency of the backbone bonds, especially from the contribution of TMPP + reporter ions.

The fidelity of producing a subsequent mass-triggered MS2 scan may be affected by data-dependent criteria in which a decision for a subsequent scan is made based on the overall intensity of the reporter ion and the AGC settings for a precursor ion^21^. For example, N-terminal labeled peptides FNWYVDGVEVHNAK and VVSLTVLHQDWLNGK produced lower intensity TMPP + diagnostic ions. However, we observed mass triggering only for FNWYVDGVEVHNAK (Fig. 3c). When we examined subsequent MS1 intensity, peptide VVSLTVLHQDWLNGK fell below the threshold for MS/MS **(**Fig. 3b**).** We also observed interesting dissociation behavior based on the sequence composition of the derivatized peptides. A single residue extension of the position of the TMPP label showed a significant effect on the gas-phase dissociation of TMPP + as a charged species. For example, VVSLTVLHQDWLNGK derivatized at both the N-terminus and the lysine side chain (Fig. 3d) generated a dominant ETD reporter ion and a triggered MS2-CID spectrum. We also observed that VVSLTVLHQDWLNGKE (Fig. 3e) with addition of a C-terminal glutamic acid and TMPP at the N-terminus produced an abundant diagnostic reporter ion and a triggered MS2-CID spectrum. We hypothesized that the propensity to generate reporter ions via ETD depends on factors such as the position of the TMPP label, amino acid composition, and charge of the peptide. Synthetic peptide data suggest that TMPP labeling occurs mostly at the N-terminus with tyrosine and lysine derivatization occurring to a lesser extent (i.e., 14 of the 15 N-termini, 1 of the 6 tyrosine residues and 1 of 10 lysine residues are TMPP labeled). Abello et al. reported that the solvent accessibility of polar residues of intact proteins can cause undesired labeling of lysine and tyrosine residues^36^. However, our triggered MS2 approach facilitates the localization on TMPP modifications effectively and promotes unambiguous determination of clip sites. ETD-MS2 exclusively localized TMPP-labeled lysine-containing peptides unambiguously on the N-terminus, and diagnostic ion-triggered CID-MS2 data complemented the ETD spectra and confirmed localization. Most of the TMPP-labeled tyrosine-containing peptides also were localized by ETD-MS2, and ion-triggered CID-MS2 spectra confirmed localization.

However, ETD-MS2 alone can be uninformative when sequence ions are insufficient to localize the site of TMPP modification. Figure 3F shows the annotated ETD-MS2 spectrum of single TMPP-labeled peptide VYACEVTHQGLSSPVTK; the search engine incorrectly assigned TMPP modification on the N-terminus. Based on the c- and z-type ETD ions, the TMPP moiety could not be unambiguously assigned to either the N-terminus or tyrosine side chain. The utility of our subsequent triggered MS2-CID scan is a confident y16 ion that unambiguously localizes the TMPP on the tyrosine residue. Figure 3G shows ETD-MS2 spectrum of a single TMPP labeled peptide EPQVYTLPPSR that exclusively produced the diagnostic ion with no backbone sequence ions. Sequence identification was based on the m/z of the precursor ion of the MS1 spectrum, whereas the reporter ion indicated that the peptide carried a TMPP moiety. The triggered MS2-CID spectrum generated several backbone ions, y7, y8, and y10, that lacked TMPP modification together with b1–b4 ions that had a TMPP moiety at the N-terminus. Note that only a few of all the MS2-CID spectra showed diagnostic ions at *m/z* 573 with limited diagnostic utility. The complementary use of MS2-ETD and triggered CID-MS2 rapidly screens potential clipped species irrespective of the amino acid sequence of a surrogate proteolytic peptide containing the TMPP moiety. Our tandem MS approach is amenable to MS2-ETD of peptides having various lengths and charge states and even peptides as small as doubly charged dipeptides. The ability to produce a dominant diagnostic TMPP immonium ion is critical for ETD-MS2 generated reporter ions to trigger a subsequent CID scan. ETD-MS2 generates TMPP diagnostic ions consistently, and both ETD and CID backbone fragment ions localize the TMPP modification to a single residue.

### Diagnostic ions of TMPP-labeled synthetic peptide derived from cell lysate

Next, from a pool of tryptic peptides and their TMPP derivatives derived from a K562 cell lysate, we assessed the diagnostic efficacy of TMPP reporter ions generated by ETD- and HCD-type fragmentation. The complexity of peptides required changes to the overall LC separation time; thus, peptides were subjected to two single-shot 6 × longer run times (120 min) with ETD-MS2 and HCD-MS2 dissociation performed separately for each run. Fig. S3A shows peptide intensities as a function of observed retention times overlaid with the AcCN gradient. The unlabeled peptides eluted up to ~ 30% AcCN, as expected. The TMPP-labeled peptides eluted adjacent to the two rapid organic ramps (0–85% AcCN) like the shorter gradient runs with TMPP-labeled synthetic standard peptides. The higher overall density of intensity distribution at the 50–70 min time window (Fig. S3B) is indicative of increased hydrophobicity and improved ionization of the TMPP labeled peptides. Fig. S3C-D shows the time distributions of the subset of TMPP-labeled peptides that had a corresponding unlabeled peptide. We next observed the time difference between the labeled and corresponding unlabeled peptides (Delta time) as function of the retention time overlaid with the AcCN gradient. The TMPP-labeled sequence eluted later for almost every peptide as indicated by large positive values (Fig. S3E-F). The density of TMPP-labeled peptides is seen at high retention times at the two rapid AcCN ramps of the gradient and at high delta time.

We next carefully evaluated the propensities to generate diagnostic TMPP reporter ions (*m/z* 533, and *m/z* 590) in the ETD spectra and TMPP reporter *m/z* 573 for HCD spectra for peptides having different lengths, amino acid composition, charge state, and sites of TMPP labeling. For every TMPP-labeled peptide, we used several different methods to estimate the efficiency of generating diagnostic ions. The reporter ion intensity was normalized to various types of product ion intensities (the relations in Eq. 1–Eq. 3 (Fig. S1)) for both types of ETD-derived reporter ions: TMPP + and TMPP-AC-NH2 + . Analogous to ETD reporter ion estimation in Eq. 1, reporter ion derived from HCD was normalized to product ions Eq. 6 (Fig. S4) For almost all peptides, TMPP + reporter ion abundance was predominant over TMPP-Ac-NH2 (Supplement Worksheet 3). In addition, we examined the overall ETD efficiency for all backbone reporters and c-, z-type product ions (Eq. 4)^35^ and all backbone fragments except the reporter ions (Eq. 5). Fig. S5A-C shows the TMPP + (*m/z* 533) efficiency as a function of peptide precursor mass or peptide length grouped by the charge state of the precursor. It was important to note a charge state dependency on the ETD efficiency for TMPP + reporter ion. TMPP + efficiency was highest in doubly charged precursor ions that decreased linearly with the mass of the peptide (Fig. S5D) while TMPP-AC-NH2 + efficiency showed a weak correlation with the mass. Also, TMPP + efficiencies were significantly higher than the efficiencies of TMPP-Ac-NH2 + (*m/z* 590) Fig. S5F. In general, the propensity to produce TMPP + reporter ions favored doubly charged precursor over triply charged precursor ions for peptides with similar mass or same number of amino acids. This observation is interesting because backbone c- and z-type fragment ions of these same peptides showed increased efficiency with increased charge states (Supplement Worksheet 3). These observations are consistent with a mechanistic study performed on doubly charged TMPP derivatized di-peptides undergoing C-P bond dissociation over N–Cα bond dissociation.^19^ We further evaluated overall backbone ETD efficiency of TMPP-labeled peptides considering all backbone fragment ions except the reporter ions. Fig. S6A-B shows the overall ETD efficiency in which considering all product ions showed a subtle charge state-dependent decrease. In contrast, Fig. S6C-D shows overall backbone efficiency estimated by Eq. 5, in which we disregarded TMPP + reporter ions. The ETD efficiency showed a charge state-dependent increase that was observed generally for unmodified tryptic peptides as reported.^32^ Considering all these observations, the contribution of TMPP + reporter ion intensities to the overall efficiency estimates, especially for doubly charged ions, was significant. The diagnostic utility of ETD-generated TMPP + ions is perfectly suited to tryptic peptides that are mostly doubly charged. In, Fig. S7A-B (and Supplement Worksheet 4), we report the HCD-derived TMPP-Ac + (*m/z* 573) efficiency as a function of peptide precursor mass grouped by the charge state of the precursor. Overall, the efficiency of the HCD generated TMPP-Ac + reporter ion was significantly lower compared with the ETD-generated TMPP + reporter ions. The efficiencies of TMPP-Ac + reporter ions of most peptides were < 1% with a significant fraction having no diagnostic reporter ions (zero efficiency). A few peptides showed extreme efficiencies as high as ~ 30% (two outliers close 60% efficiency were false positive assignments in which the precursor m/z was the same as reporter ion m/z). We did not observe a charge state-dependency on the HCD efficiency for the TMPP-Ac + reporter ion.

Next, we evaluated the likelihood of TMPP off-labeling of lysine and tyrosine residues. We identified 12 peptide-to-spectrum matches (PSMs) of TMPP-labeled tyrosine residue from a total of 520 tyrosine-containing PSMs. From these 520 tyrosine-containing PSMs, 262 had TMPP derivatized N-termini and the remaining 246 were unmodified. No PSMs of TMPP-labeled lysines were identified from 1441 lysine-containing PSMs. Of the 1441 lysine-containing PSMs, 771 PSMs had TMPP-derivatized N-termini, 11 had TMPP-derivatized tyrosine, and the remaining 659 were unmodified (Supplement Worksheet 3). Fig. S8A-B shows that the ETD efficiency had no effect on the labeled peptides grouped by the number of tyrosine and lysine residues per peptide. These data suggested that the TMPP labeling of peptides was mostly at N-termini under our reaction conditions, and additional unlabeled lysine or tyrosine residues had no effect on diagnostic TMPP + ion. Fig. S9 shows the overall distribution of the reaction or TMPP labeling efficiency of peptides estimated by Eq. 7. The derivatized precursor ion subjected to ETD had no bearing on the levels of derivatization, i.e., a peptide derivatized at 100% or 1% would have the same ETD efficiencies. This is important when considering these reactions in the context of clip site identification of proteins; derivatization efficiency has no consequence on the ETD efficiency of the surrogate peptide.

**Figure 4:**
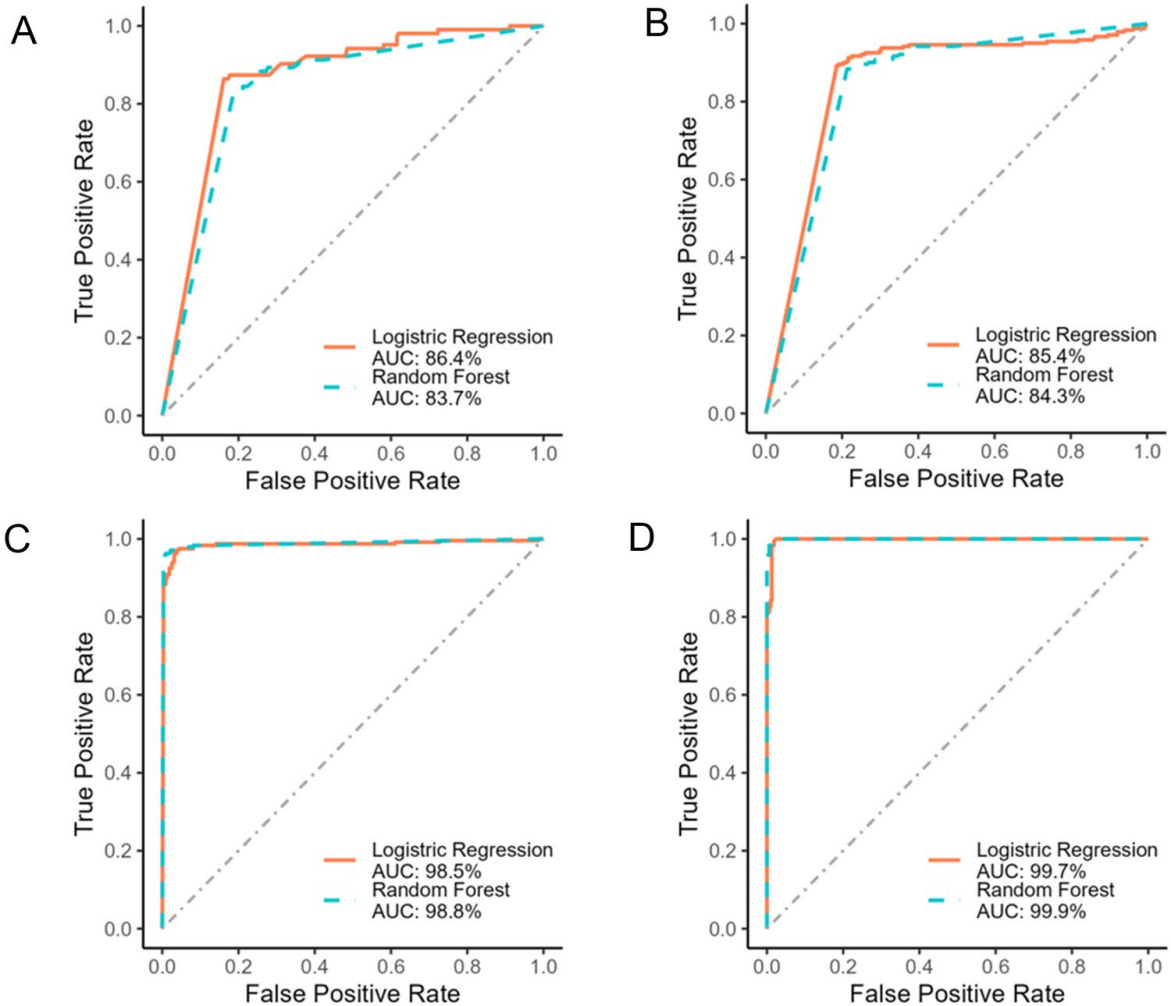
Receiver Operator Characteristic (ROC) curves obtained via logistic regression (lr) and random forest (rf) to model diagnostic ions and the elution time features of TMPP labeled peptides derived from K562 digest. (**A**) HCD-MS2 derived TMPP-Ac + (AUC, lr = 86.4%, rf = 83.7%) (**B**) ETD-MS2 derived TMPP-Ac-NH2 (AUC, lr = 85.4%, rf = 84.3%) (**C**) ETD-MS2 derived TMPP + (AUC, lr = 98.5%, rf = 98.8%) (**D**) Observed retention time of TMPP labeled peptides (AUC, lr = 99.7%, rf = 99.9%).

Finally, we examined the diagnostic utility of each reporter ion generated by ETD and HCD and LC retention time. TMPP-derivatized peptides and their unmodified counterparts were confidently identified via searches against the human protein sequences, with sequence ions localizing the TMPP moiety with high confidence on mostly peptide N-termini. We used the search results to determine and separate the classes of TMPP-labeled peptides from unlabeled peptides. We then used the dissociation efficiency (ETD or HCD) of the diagnostic ion for each PSM where TMPP-labeled identifications and unlabeled identifications were grouped as true and false respectively. These selections were then subjected to logistic regression and random forest algorithms to evaluate the performance of each model. After training with tenfold cross validation on 75% of the dataset, receiver operating characteristics (ROC) curves were generated for both ETD and HCD diagnostic ions as well as for retention time of labeled and unlabeled peptides to visualize sensitivity and specificity of the classification models. Fig. S10A-C illustrates how search results and diagnostic ion abundance were used to classify or misclassify spectra. The labeled peptide that produces a characteristic TMPP + reporter ion is a True Positive (TP), whereas the unlabeled peptide counterpart does not produce a reporter and is a True Negative (TN). In, Fig. S10B, the labeled peptide that produces a characteristic TMPP + reporter ion is a True Positive (TP), whereas the unlabeled peptide counterpart produces an interfering ion similar in mass to the TMPP + reporter ion and is a False Positive (FP). In Fig. S10C, the modified peptide does not produce a diagnostic ion and is a False Negative (FN), whereas the unmodified peptide counterpart produces an interfering ion similar in mass to the TMPP + reporter ion and is a False Positive (FP). Likewise, we used TMPP-Ac-NH2 + , TMPP-Ac + and retention time of TMPP labeled peptides to obtain accuracy and precision of the features. Figure 4 shows the Area Under the Curve (AUC) of the Receiver Operating Characteristic (ROC) curve for each diagnostic ion and the elution time. Of the reporter ions, TMPP + reporter ions were the most diagnostic with an AUC of ~ 98%, followed by TMPP-Ac-NH2 + (AUC ~ 84–85%) and TMPP-Ac + (AUC ~ 83–86%). Thus, compared with the other reporter ions, the ETD-generated TMPP + diagnostic ions were most accurate and the most specific. The ROC curve of the observed retention time AUC of ~ 99% was the most diagnostic of all measures.

### TMPP labeling and ETD reporter ion-triggered CID for detecting therapeutic protein degradation

**Figure 5:**
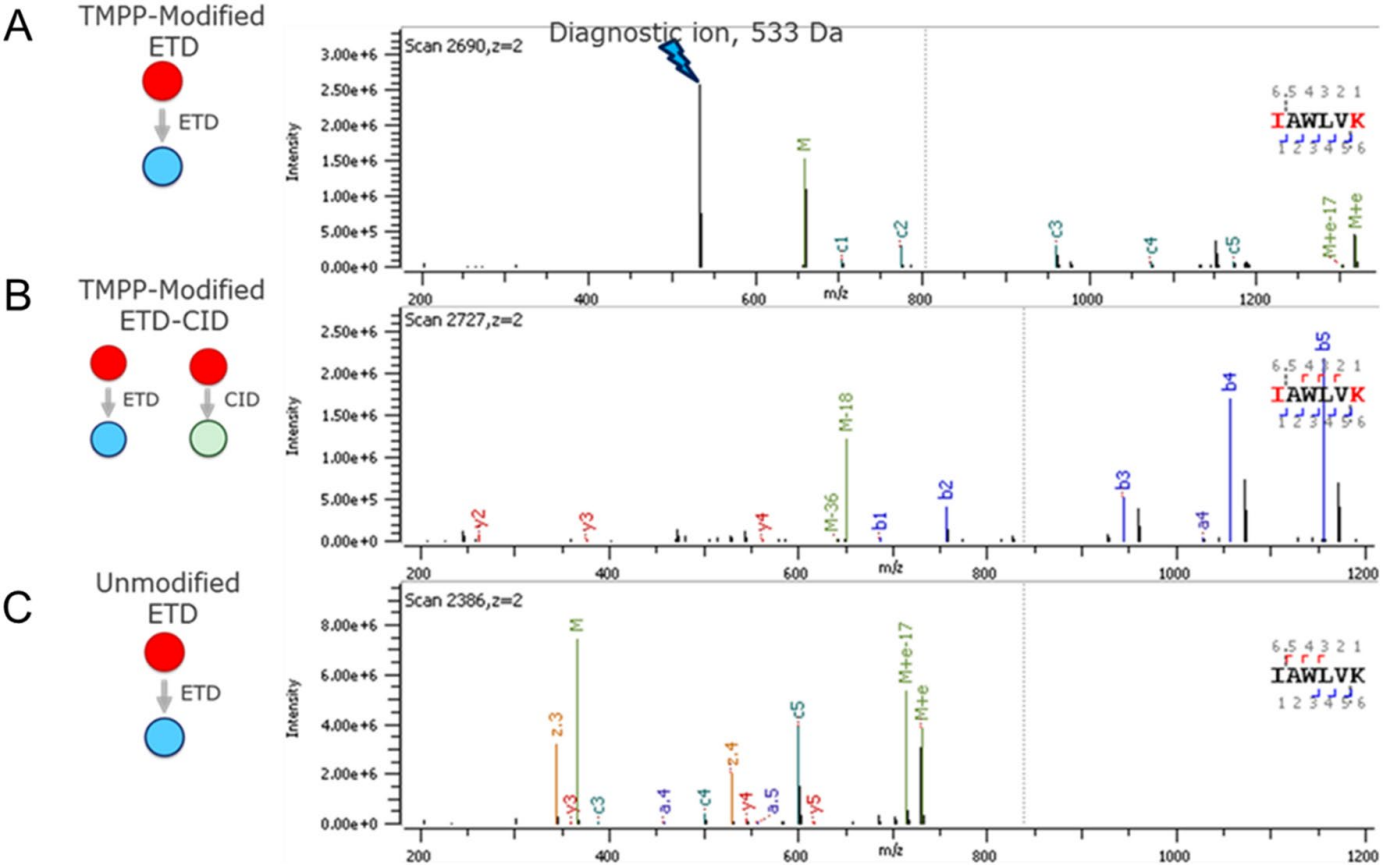
Identification of Cathepsin-induced neo-*N*-terminal clip site F-I of GLP1-Fc (**A**) ETD-MS2 of TMPP labeled (at N-terminus and Lysine) IAWLVK peptide (2 +) (**B**) TMPP + (533) reported ion triggered CID-MS2 of TMPP labeled (at N-terminus and Lysine) IAWLVK peptide (2 +) (**C**) CID-MS2 of corresponding unlabeled IAWLVK peptide (2 +) counterpart.

**Figure 6:**
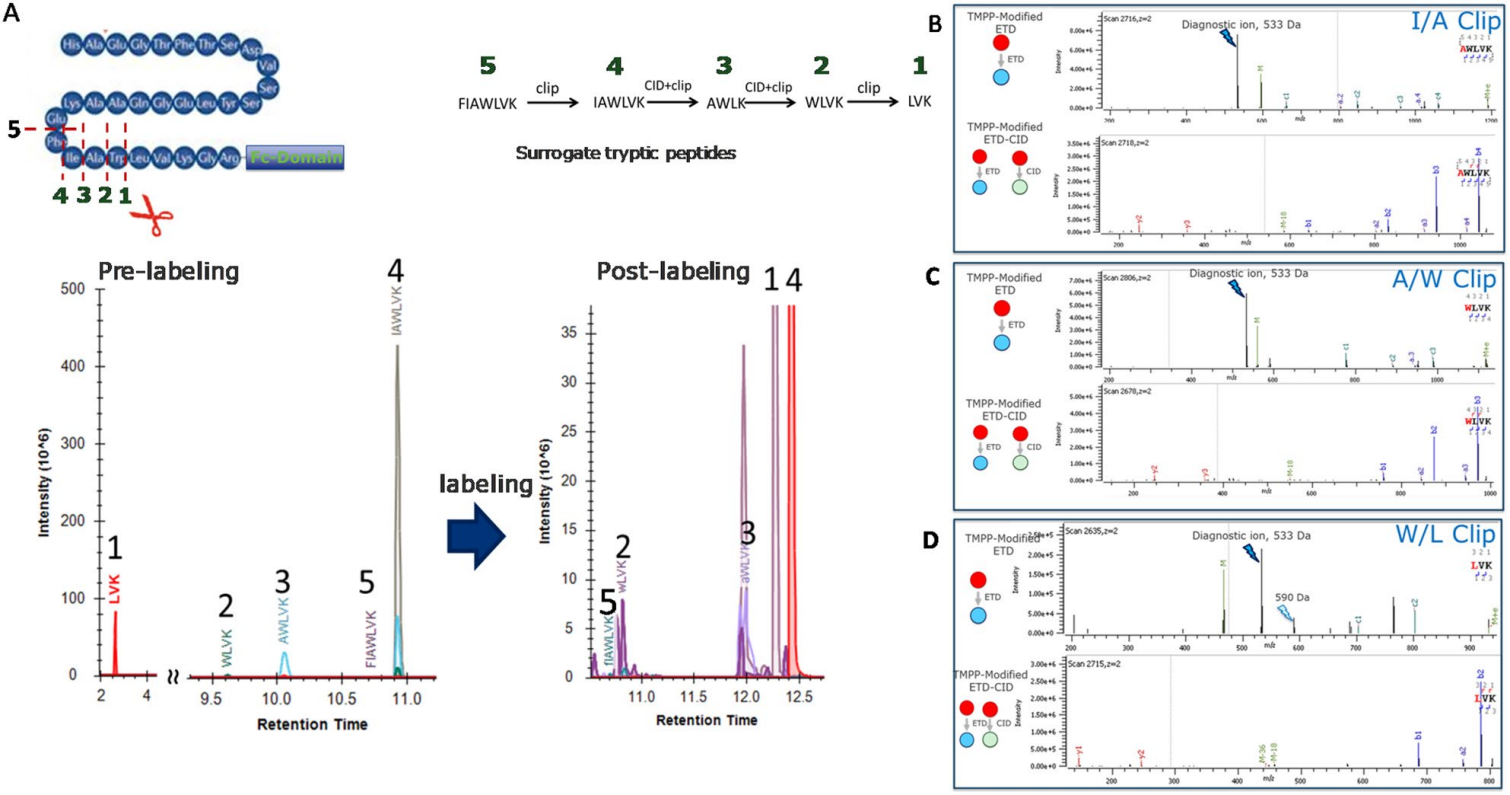
Multiple sequential Cathepsin-induced neo-*N*-terminal clip sites of GLP1-Fc (**A**) Extracted ion chromatograms of the surrogate peptides and Reporter ion Triggered Ion MS2 of TMPP derivatized clipped sites of GLP1-Fc fusion protein (**B**) I/A clip-site (**C**) A/W clip-site (**D**) W/L clip-site.

We developed the foregoing methodology particularly to detect clipping of therapeutic proteins. Here, we used a commercial preparation of Dulaglutide fusion protein, a Glucagon-like Peptide 1 (GLP1) fused to Crystalline Fragment (Fc), to assess the efficacy of our technology. Dulaglutide is known to undergo protease-mediated clipping of the GLP1 moiety. Thus, we treated Dulaglutide with cathepsin D to study putative clip sites by ETD-MS2 and diagnostic ion-triggered CID-MS2. Previous investigators reported that cathepsin D cleaves GLP1 at W25/L26^3,37,38^. Figure 5. shows the MS2 spectra evidence for neo-N-termini created by the protease. The product ion spectrum (Fig. 5a) from ETD-MS2 of the doubly charged ion produced a characteristic diagnostic TMPP + ion (*m/z* = 533). In addition, we observed c-type ions in the TMPP site localization. Site localization of TMPP on the *N*-terminus is indicative of a neo-*N*-terminus due to F-I clip, In addition C-terminus lysine was labeled with a second TMPP on that can be inferred as a K-G clip because a TMPP moiety conjugated to a lysine is resistant to trypsinization. Note that TMPP labeling can also occur on lysine and tyrosine residues, and peptides carrying TMPP modifications subjected to ETD will also generate diagnostic reporter ions, hence, triggered MS2-CID events. These CID spectra are false positive identifications of reporters. Nevertheless, careful examination of the sequence ions in ETD and diagnostic ion-triggered CID spectra can localize TMPP in the sequence and eliminate false-positives. Figure 5b shows the diagnostic reporter ion (*m/z* 533) triggered CID-MS2 spectrum of the doubly charged ions. The b- and y-type ions assist in TMPP site-localization of both the *N*- and *C*-terminal lysine residues. The same peptide sequence did not generate characteristic CID-induced reporter ions (*m/z* 573). We also generated the ETD spectrum of the unconjugated peptide (Fig. 5c) which lacks diagnostic ions. The product ion distribution of the unconjugated peptide gave a mixture of both c- and ·z-type ions. The complete analysis of Dulaglutide peptides revealed additional clipping of GLP1. Figure 6A shows the extracted ion chromatograms of the surrogate peptides 1–5 that consists of neo-*N*-termini due to clipping. These peptides when labeled with TMPP shows increased retention during RP-LC. Figure 6B–D shows further evidence of ETD-MS2 and diagnostic ion-triggered MS2-CID product ion spectra of surrogate peptides that corresponded to the sequential clipping of GLP1 sequence. The surrogate peptides that result from I/A clip generated exclusively a TMPP + (*m/z* 533) diagnostic ion, whereas those resulting from a A/W and W/L clip produced diagnostic ion TMPP + at *m/z* 533 and TMPP-Ac-NH2 + at *m/z* 590 during ETD. The predominant diagnostic ions at *m/z* 533 that triggered a MS2-CID event for each peptide generated a CID product ion spectrum. The CID-MS2 spectra complemented the ETD identifications, and the triggered MS2 scans seamlessly confirmed the presence of reporter ions generated from ETD-MS/MS to unambiguously identify neo-*N*-termini for the entire data set. In addition to CID, we assessed the utility of other dissociation modes such as HCD, which creates more internal fragments^39–43^, and UVPD, for generating reporter ions. Table S1 summarizes the results for the series of clipped sites for GLP1 surrogate peptides IAWLVK, AWLVK, WLVK and LVK. Although all these peptides generated the characteristic *m/z* 533 diagnostic ions via ETD, only the LVK peptide showed diagnostic ions for HCD and UVPD dissociation modes (Fig. S11). HCD produces a characteristic diagnostic ion at *m/z* 573 due to amide bond dissociation^44,45^, and UVPD produces a diagnostic ion at *m/z* 181 presumably due to further dissociation and rearrangement^46^. Besides not detecting diagnostic ions in every peptide, significantly lower relative peak intensities compared with the ETD-generated diagnostic ions makes triggering of the HCD and UVPD less informative. The XIC of every TMPP-labeled peptide elutes during the two rapid ramps between 10 and 13 min. The short LVK peptide observed predominantly in endogenous samples was less retentive and, presumably, elusive in peptide mapping experiments. However, the same peptide post-TMPP labeling had a significant column retention during reversed phase chromatography due to the increased hydrophobicity of the peptide. The presence of an unlabeled peptide counterpart of a TMPP labeled peptide further validates the identities of neo-*N*-termini that result from clipping.

## Conclusion

We report the use of TMPP labeling in conjunction with electron transfer dissociation (ETD) mass spectrometry to generate facile diagnostic ions of TMPP + and TMPP-Ac-NH2 + . The TMPP + reporter ion was most intense for small tryptic peptides. This observation was unusual compared with the typical backbone dissociation efficiencies that usually increase with precursor ion charge for peptides with similar lengths. ETD efficiency of a doubly charged ion was lower than triply or quadruply charged ions due to the creation of neutral product ions from a single cleavage^32,35^. We suggest that the fixed charge group on the TMPP moiety facilitates efficient electron recombination to produce a favorable fragment that retains the charge^33^. We demonstrate the various factors that affect the production of TMPP + reporter ions using synthetic standard peptides of NIST monoclonal antibody as well by generating a large pool of peptides from K562 cell lysate with various lengths, charge states, and sequence compositions. TMPP + reporter ion efficiency was highest for small doubly charged peptides. In contrast, HCD generated TMPP-Ac + reporter ions did not show charge state dependence. The diagnostic utility of ETD generated TMPP + ions was determined by the AUC of ~ 98% compared to AUC of ~ 83–86% for HCD generated TMPP-AC + ions of a ROC analysis. The generation of TMPP + ions for triggered scans enables complete interrogation of sequence for accurate localization of the TMPP moiety or to confirm a sequence with high confidence when ETD fails to generate enough backbone fragments of doubly charged ions. The high fidelity of triggered MS2 was demonstrated for a panel of TMPP derivatized NIST synthetic peptides and tryptic peptides generated from GLP1-Fc fusion protein derivatized with TMPP. N-terminal TMPP establishes the clipped site prior to digestion and mass spectrometry, both of which produce spurious fragments that can be mistaken for clipped sites. We finally demonstrate the utility of TMPP + diagnostic reporter ion-triggered MS2 to examine Cathepsin-induced clipping-sites of the GLP1. We obtained evidence of ETD-MS2 and diagnostic ion triggered MS2-CID product ion spectra of surrogate peptides corresponding to the sequential clipping of GLP1 sequence. The sequential clips were each confirmed with high confidence via TMPP + diagnostic ions and subsequent reporter ion-triggered CID-MS2. The CID-MS2 spectra complement the ETD identifications and triggered MS2 scans provides a real time *in silco* filtering mechanism in which a CID scan is performed only when the reporter ion is observed. The reporter ion-triggering provides high confidence identification and seamless assembly of neo-*N*-termini for the entire data set. This mode of analysis reduces the ambiguity of detecting clipped sites where labeling was not performed, and it obviates the need to evaluate spurious artifacts created by sample digestion and mass spectrometry conditions.

## Data availability

The MS/MS datasets generated during the current study and R script used to for ROC analysis are available in the figshare repository, https://figshare.com/projects/Diagnostic_Utility_of_N-terminal_TMPP_labels_for_Unambiguous_Identification_of_Clipped_Sites_in_Therapeutic_Proteins/176742.

### Supplementary Information

Supplementary Information 1.

Supplementary Information 2.

Supplementary Information 3.
