# Supplementary material for: Diagnostic utility of N-terminal TMPP labels for unambiguous identification of clipped sites in therapeutic proteins
Source: Sci Rep. 2023 Oct 30;13:18602. doi: 10.1038/s41598-023-45446-z (PMC10616084; doi:10.1038/s41598-023-45446-z)
Supplement: Supplementary file 1 — Supplementary Information 1. [file 41598_2023_45446_MOESM1_ESM.docx]

Supporting Information

Diagnostic Utility of N-terminal TMPP labels for Unambiguous Identification of Clipped Sites in Therapeutic Proteins

Harsha P. Gunawardena^1^* Meth M. Jayatilake^2^, Jeffery D. Brelsford^1^, Hirsh Nanda^1^

1. Janssen Research and Development LLC, The Janssen Pharmaceutical Companies of Johnson & Johnson, Spring House PA, USA

2. Department of Oncology, Lombardi Comprehensive Cancer Center, Georgetown University Medical Center, Washington DC, USA.

*Correspondence: [hgunawar@its.jnj.com](mailto:hgunawar@its.jnj.com)

Table of Contents

Figure S1............................................................................................................................................. S-2 Figure S2............................................................................................................................................. S-3 Figure S3............................................................................................................................................. S-13

Figure S4………………………………………………………………………………………………………………………………………. S-14

Figure S5………………………………………………………………………………………………………………………………………. S-14

Figure S6............................................................................................................................................. S-15

Figure S7............................................................................................................................................. S-16 Figure S8............................................................................................................................................. S-16 Figure S9............................................................................................................................................. S-17 Figure S10........................................................................................................................................... S-18

Figure S11........................................................................................................................................... S-19

Table S1………………………………………………………………………………………………………………………………………… S-20 References.......................................................................................................................................... S-20


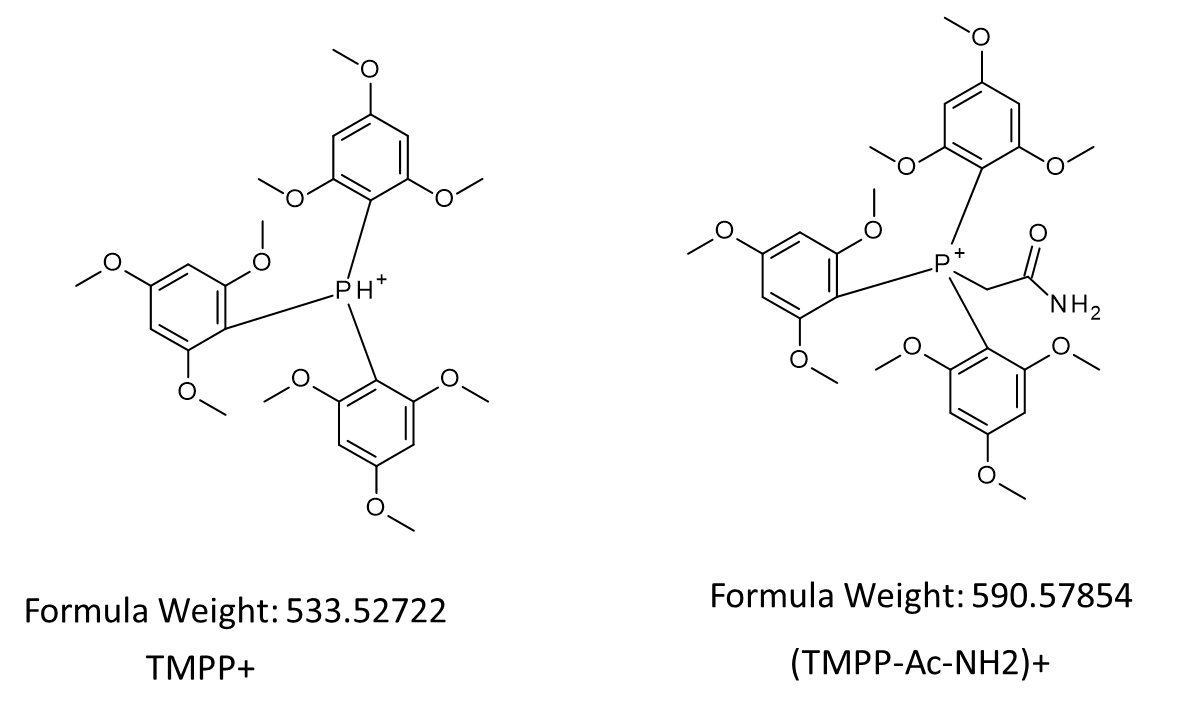

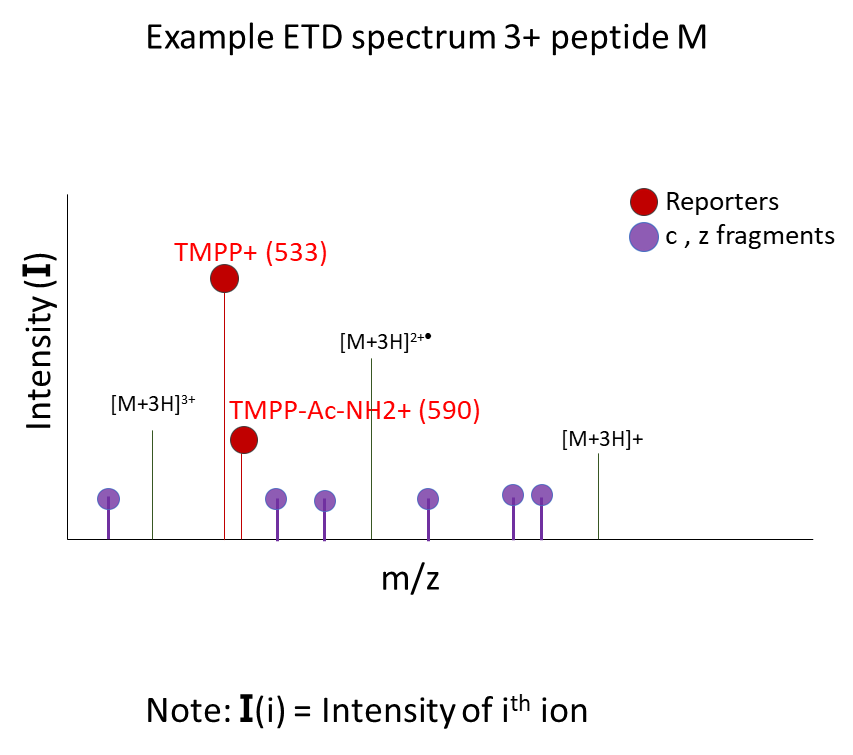


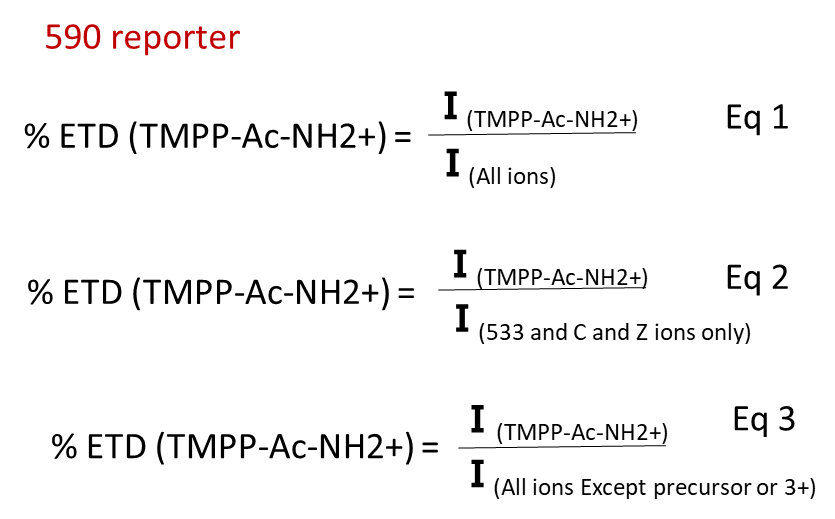

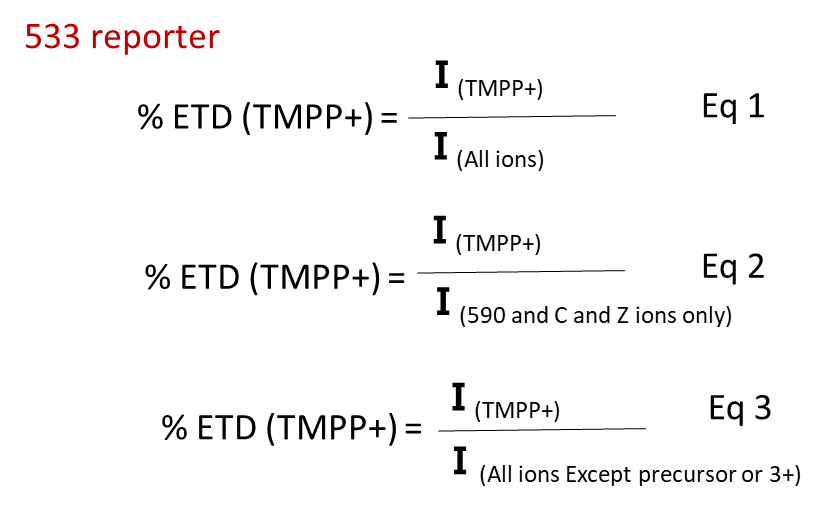


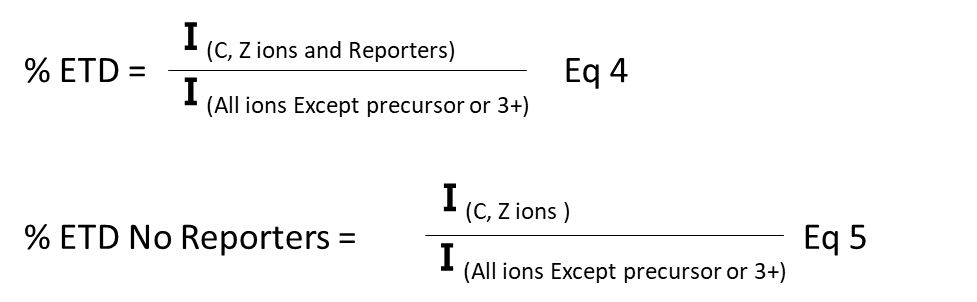


Fig S1. Electron transfer dissociation (ETD) efficiency estimated using TMPP derived reporter ions: TMPP+ and TMPP-Ac-NH2+ and peptide backbone c- and z-type fragment ions. Eq1-Eq5 are %ETD efficiency estimates using Intensities (I) of the reporter ions, backbone fragment ions and unfragmented, charge reduced precursor ions: [M+3H]3+•, M+3H]+••.

| 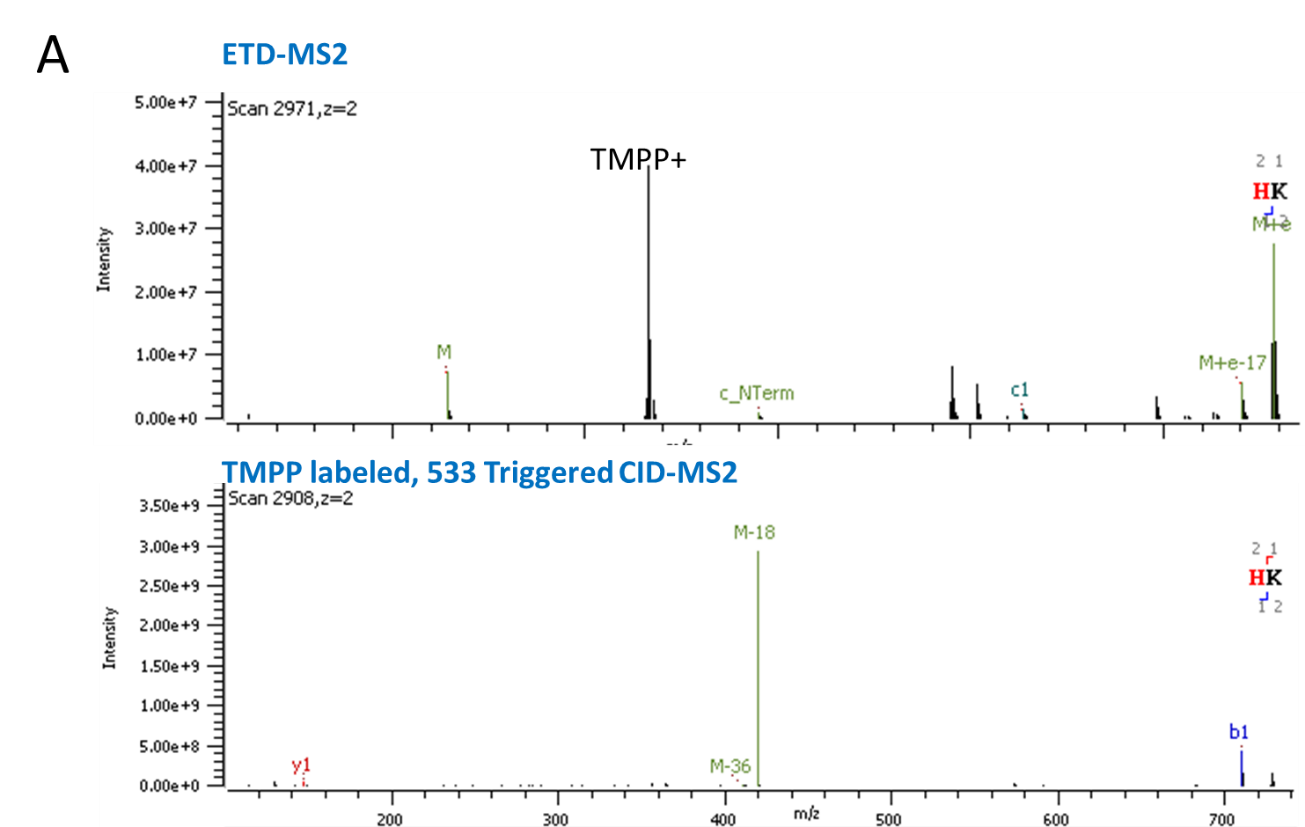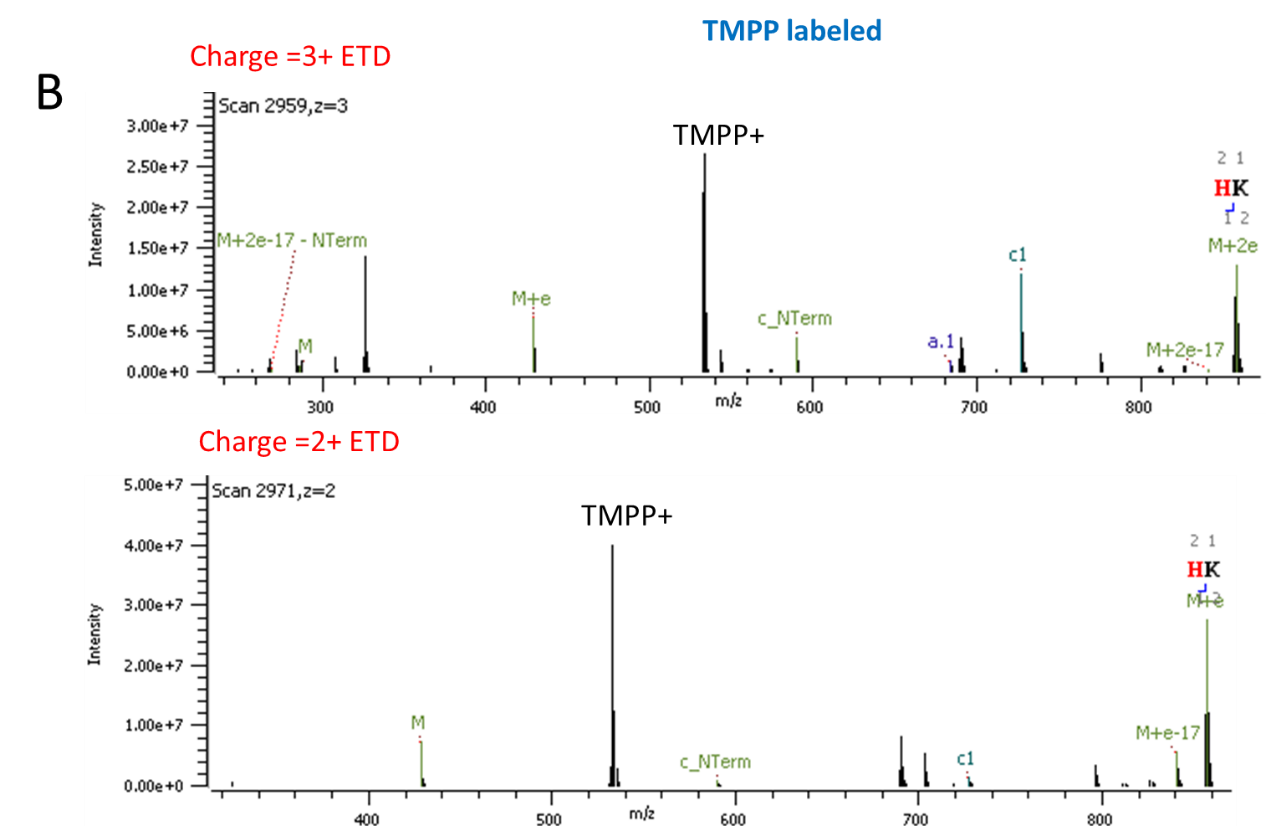 |
| --- |


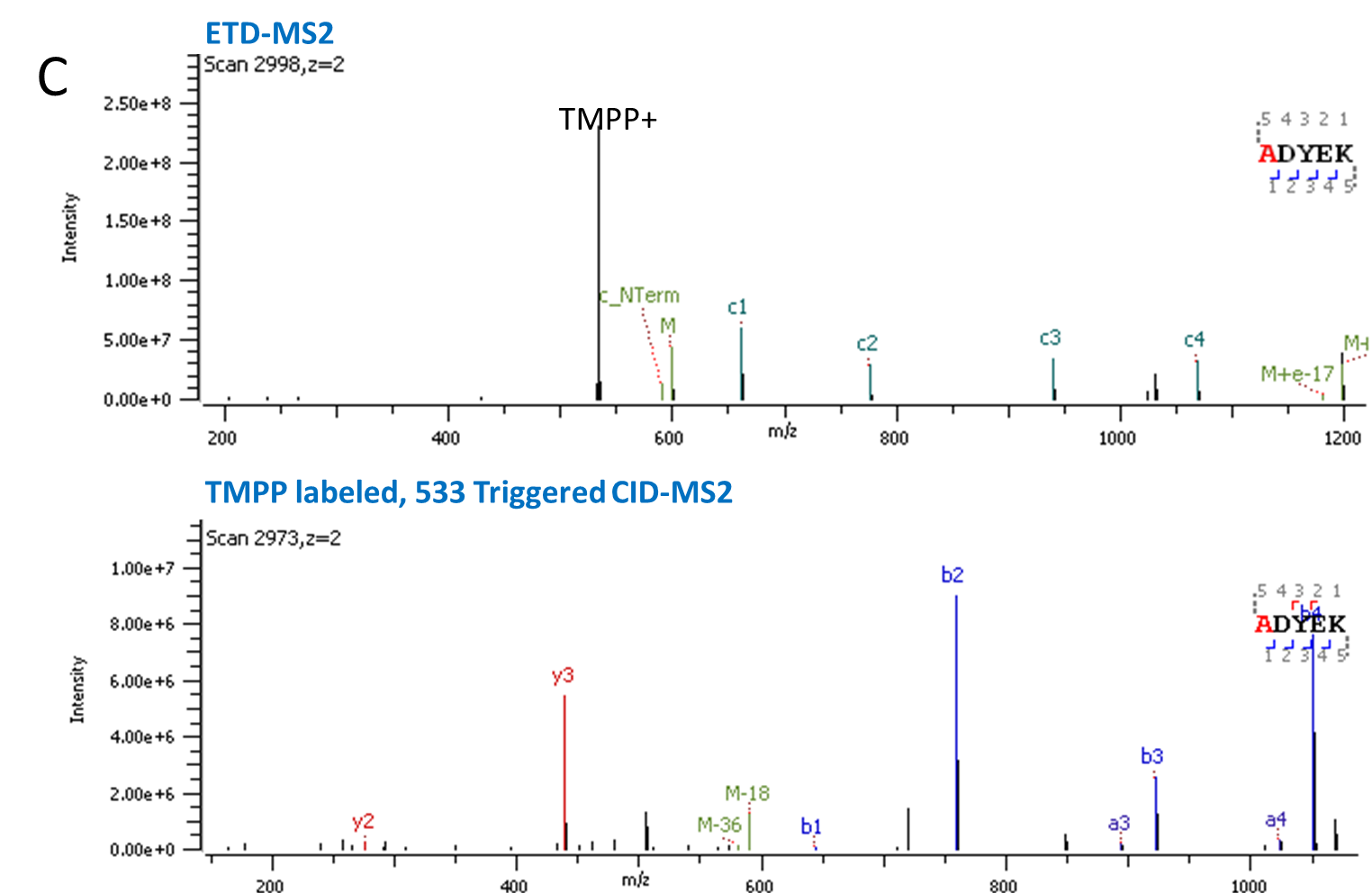


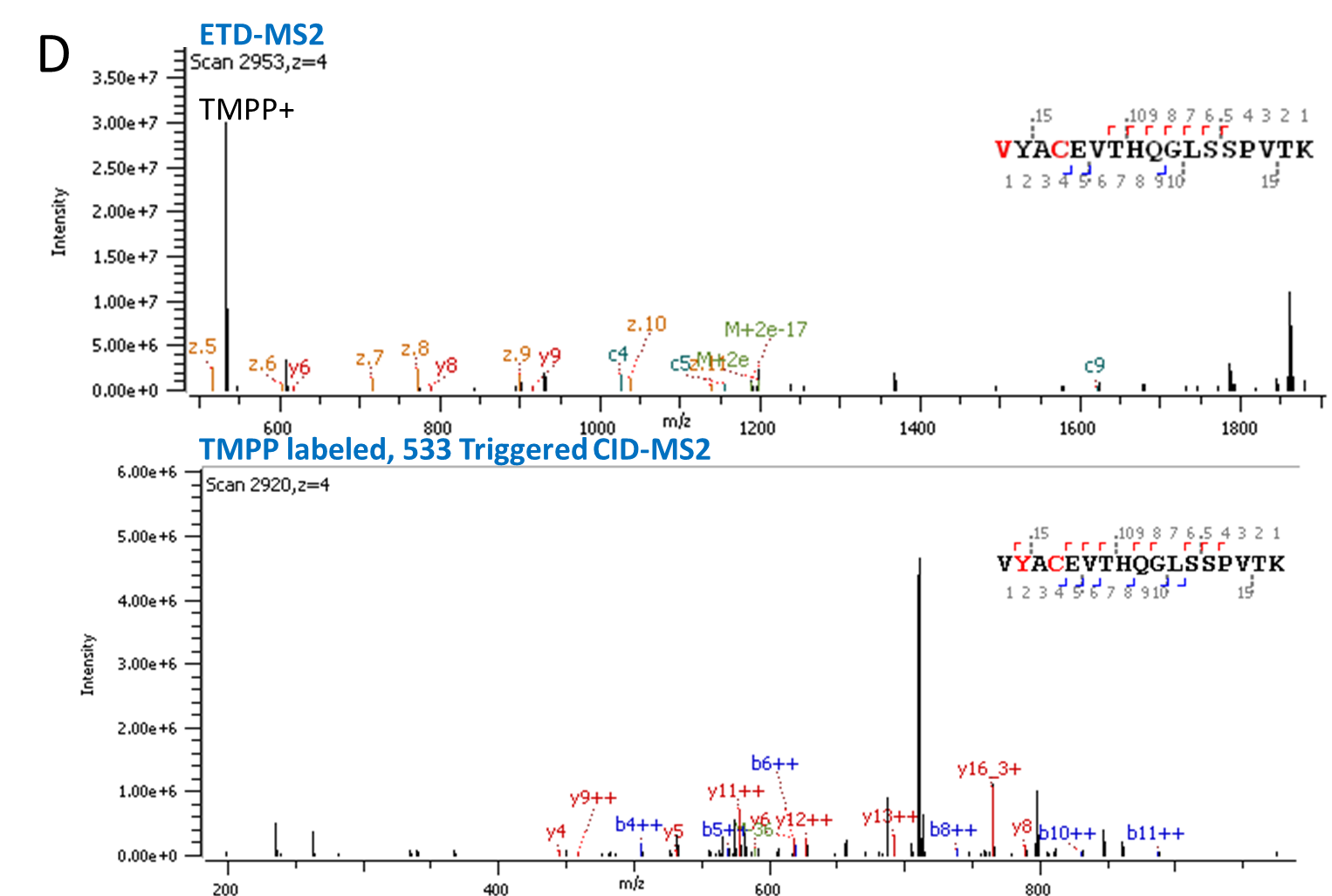


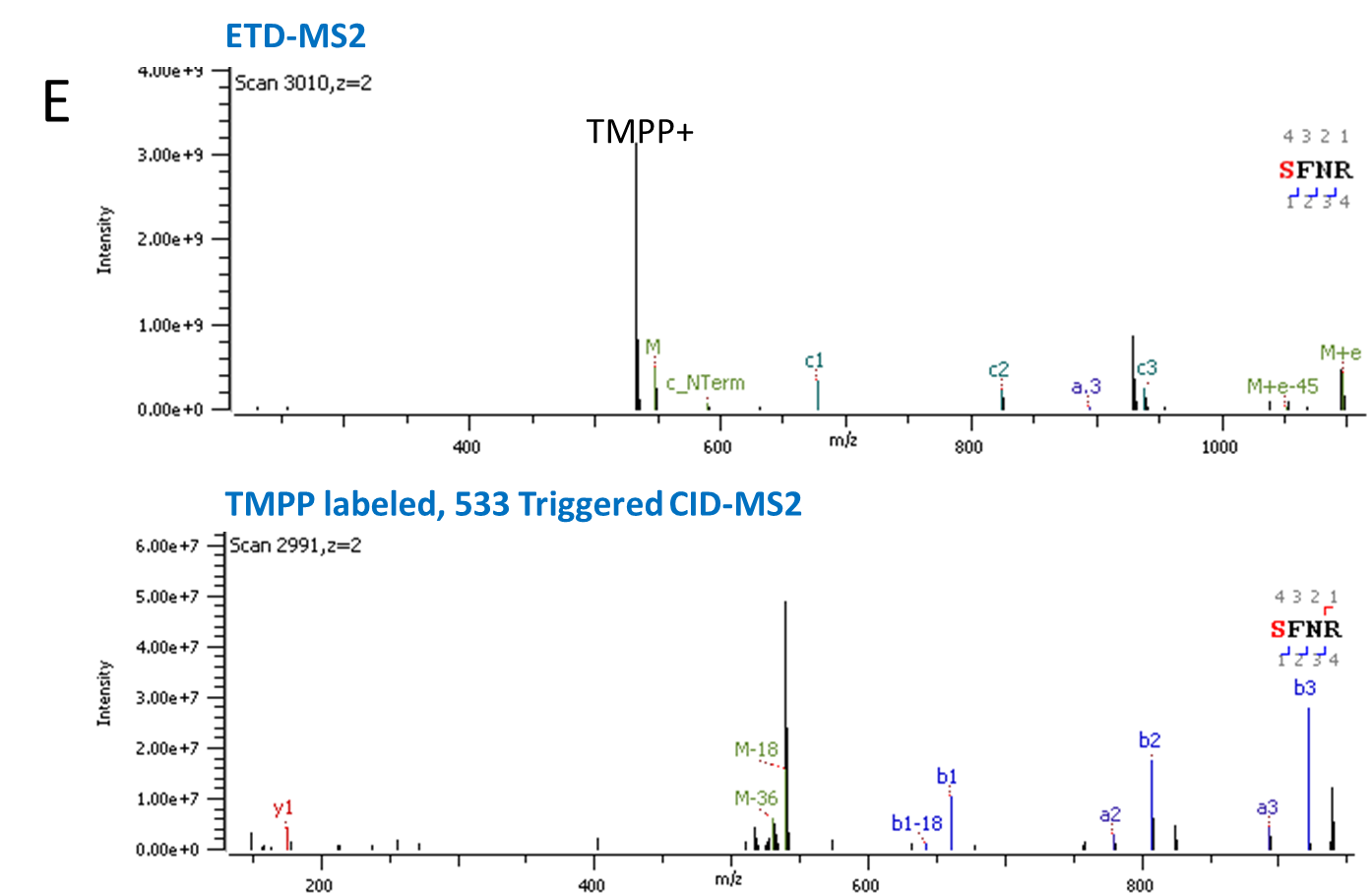


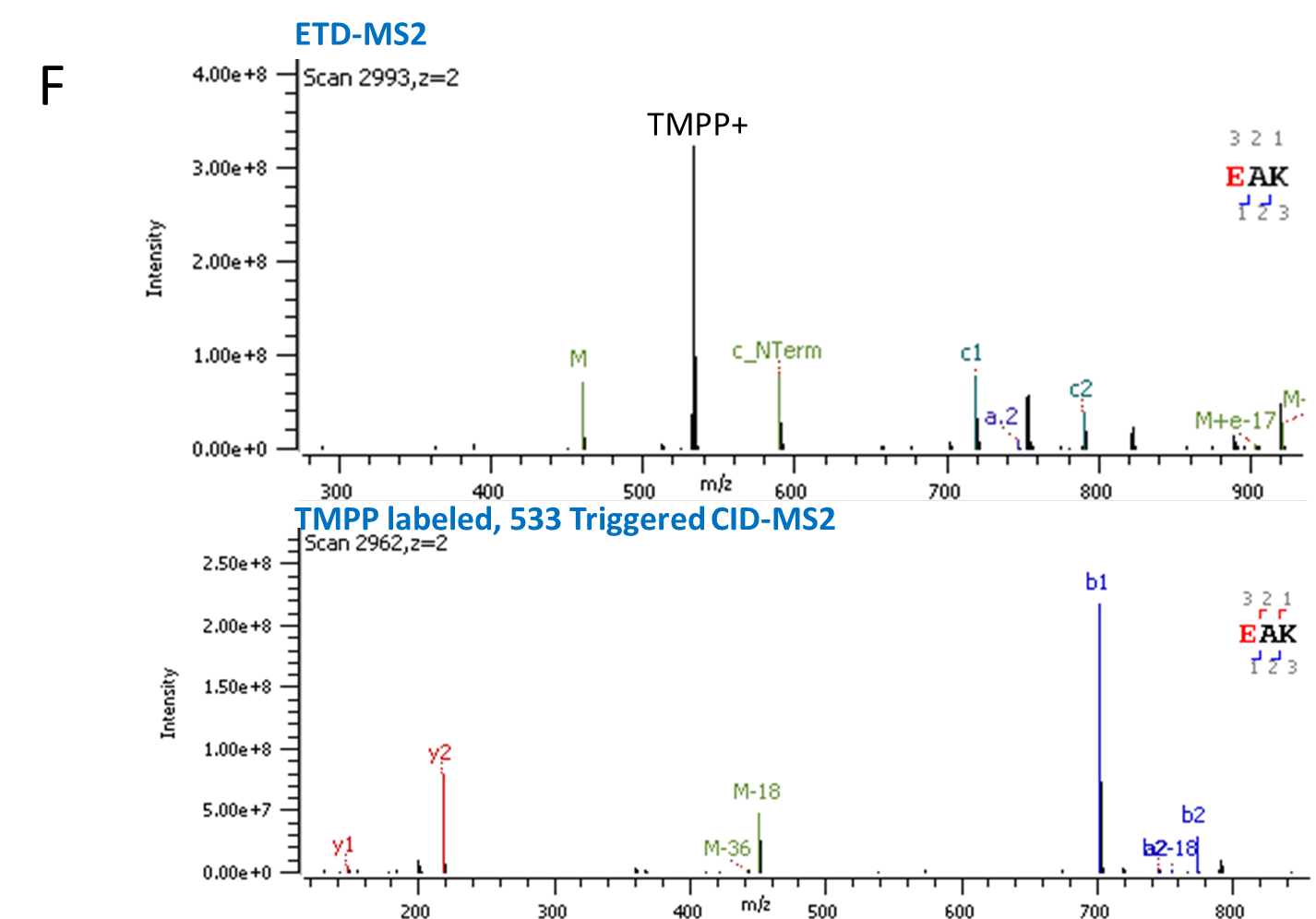


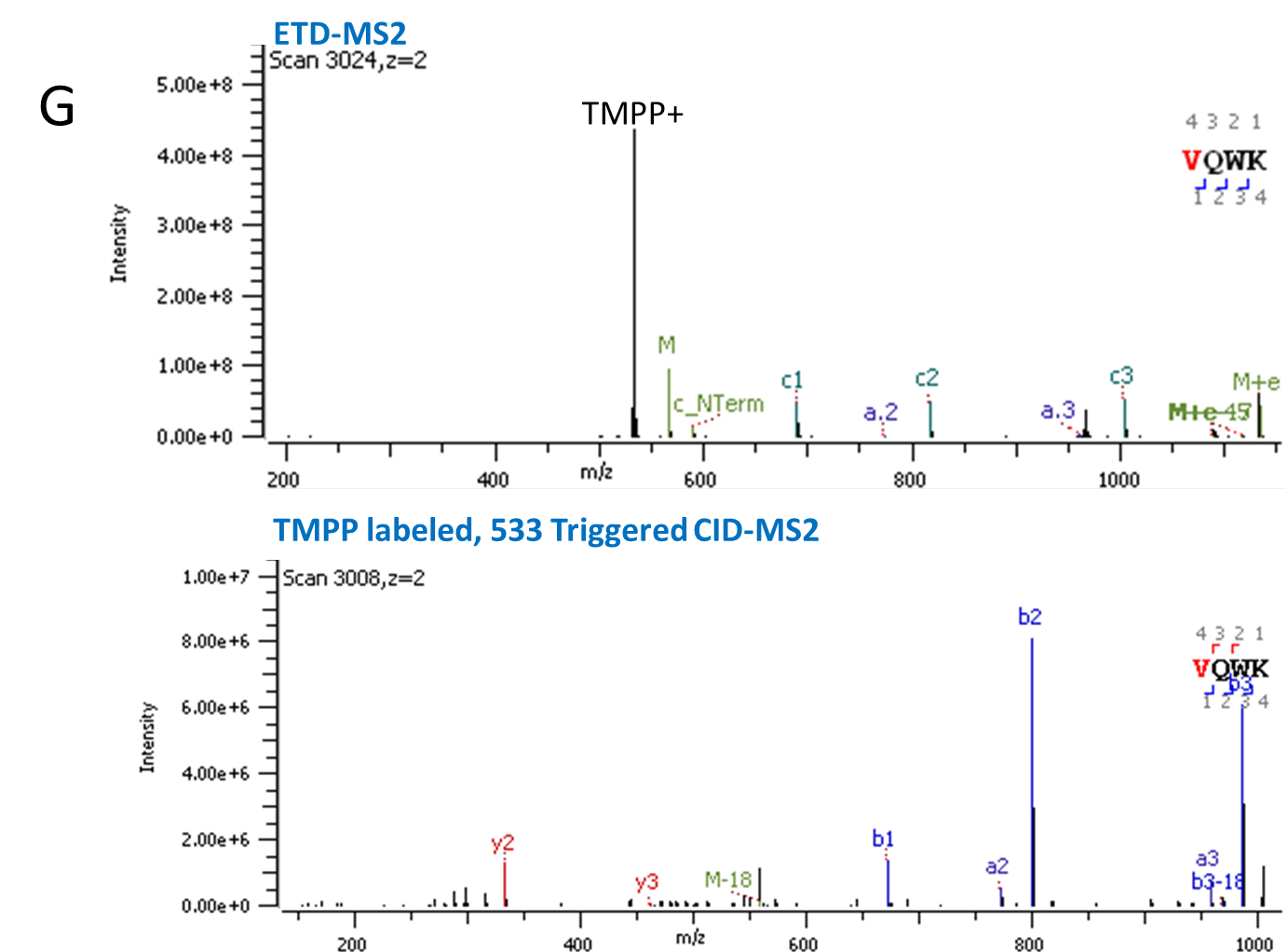


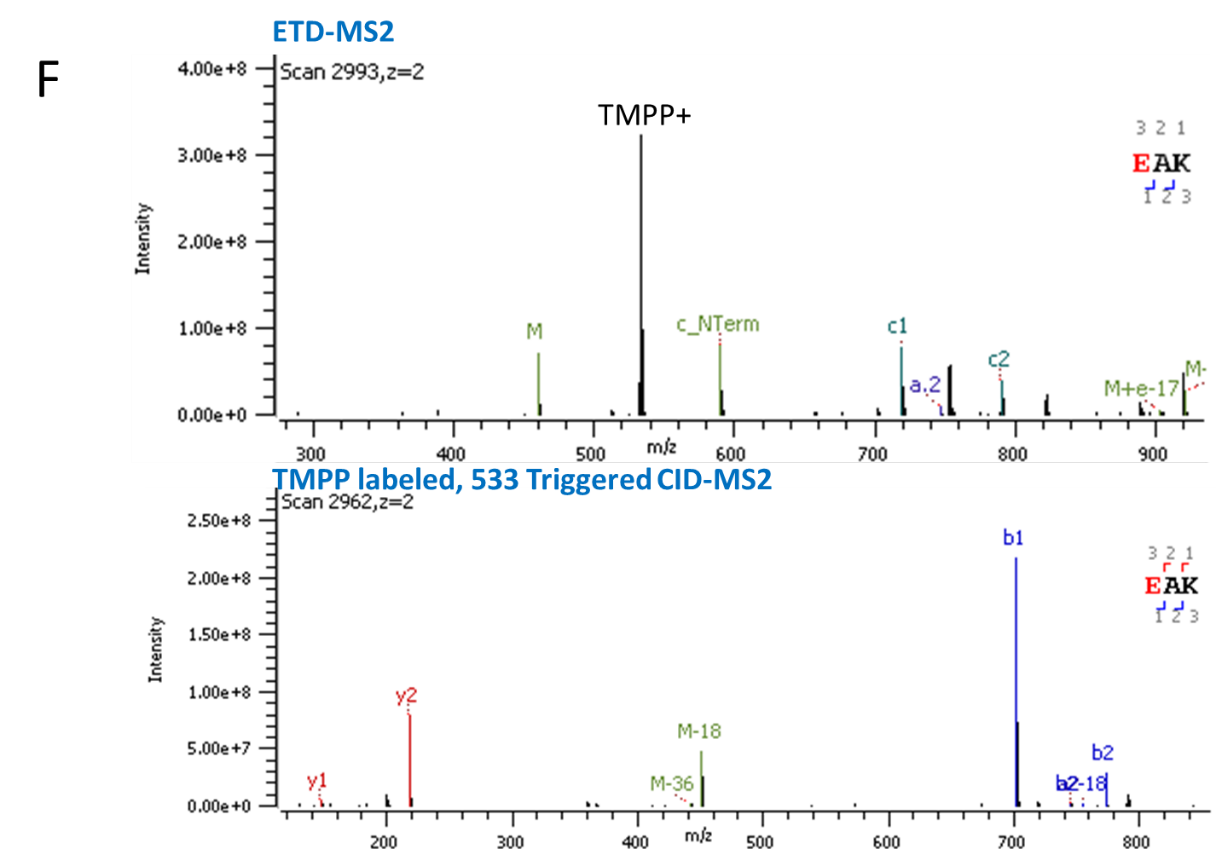


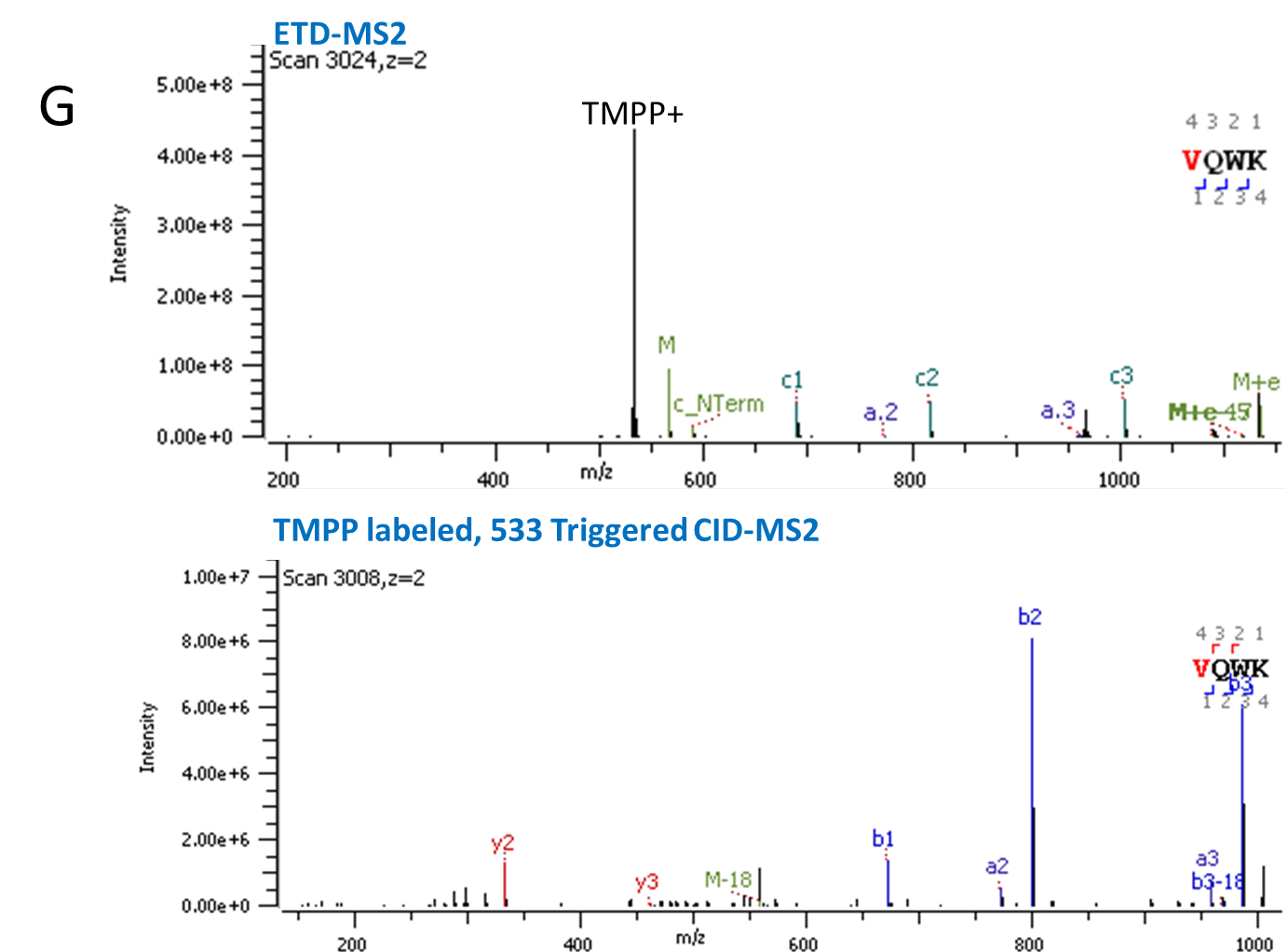


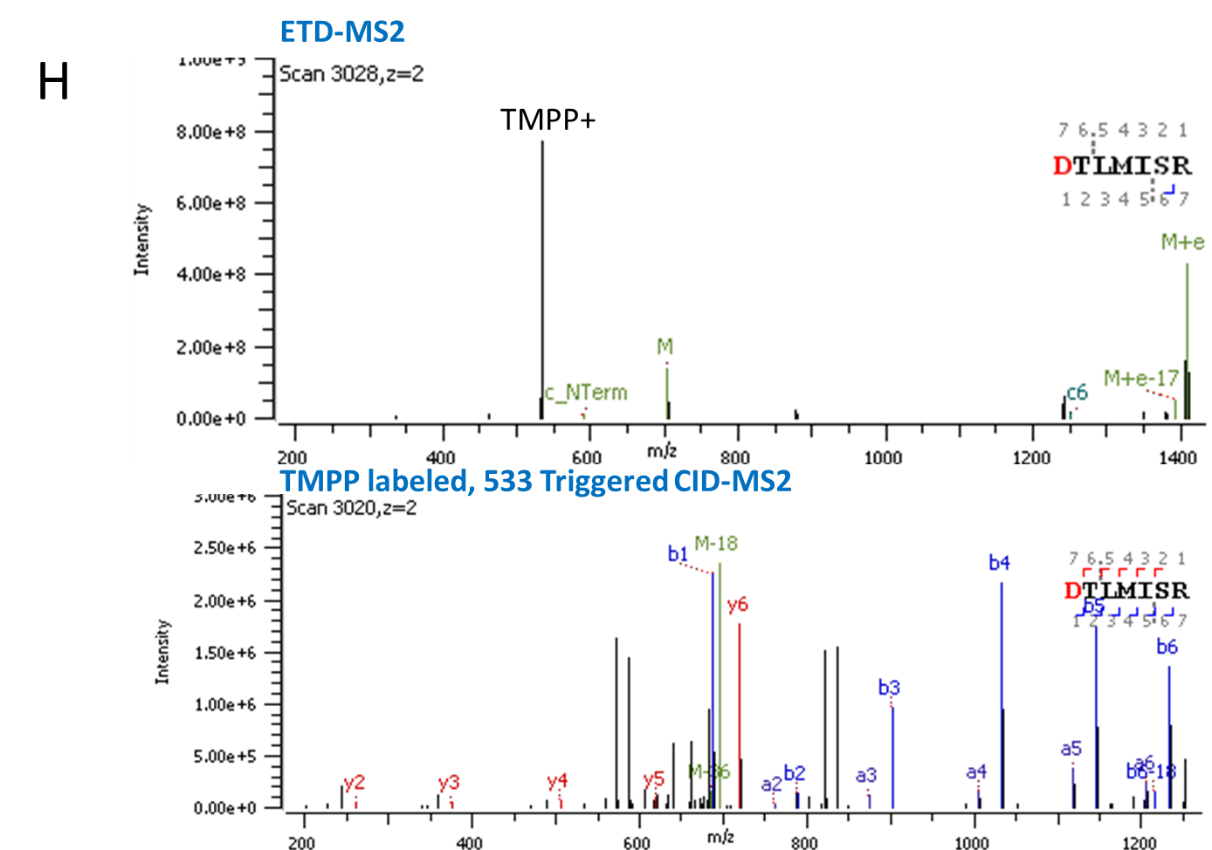


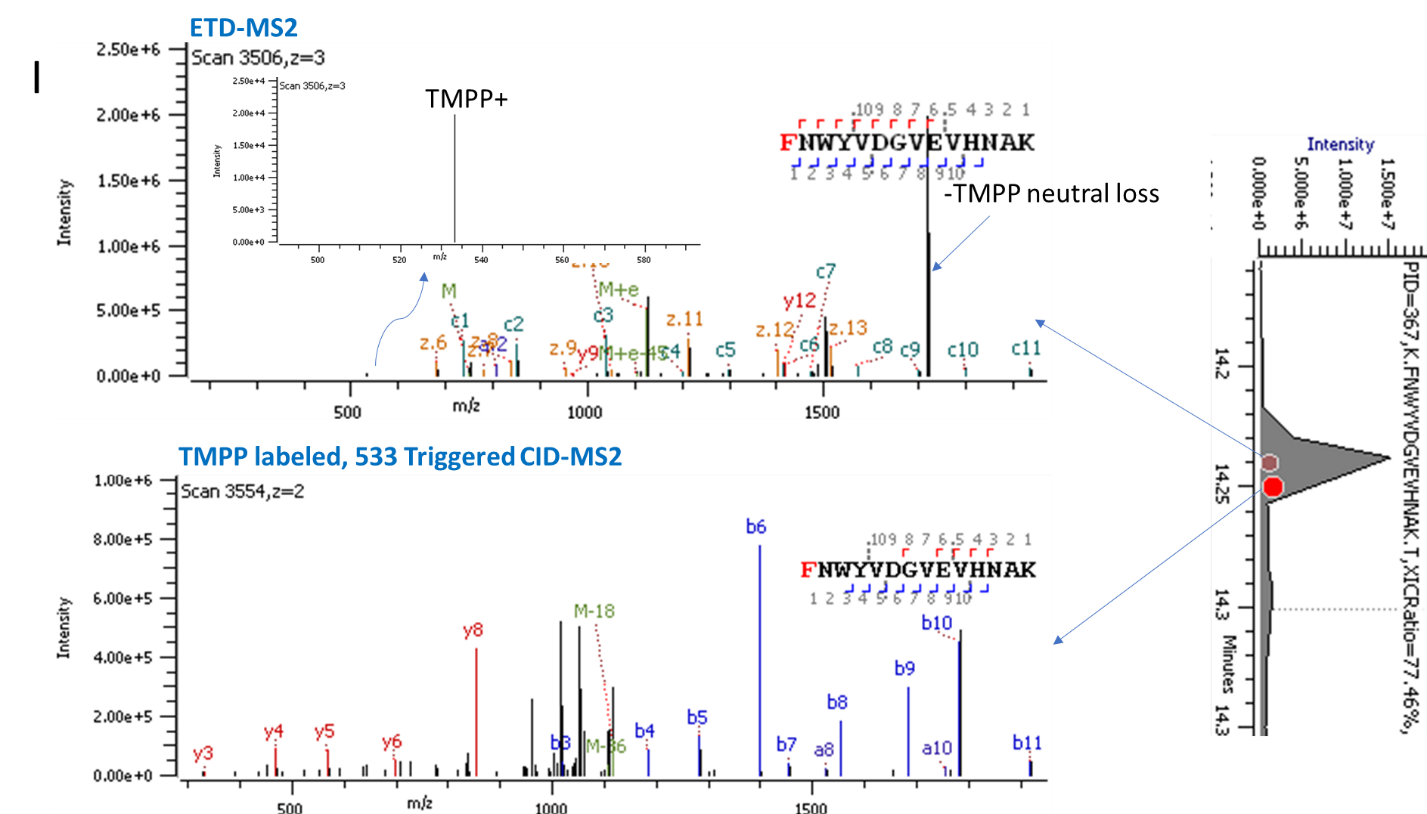


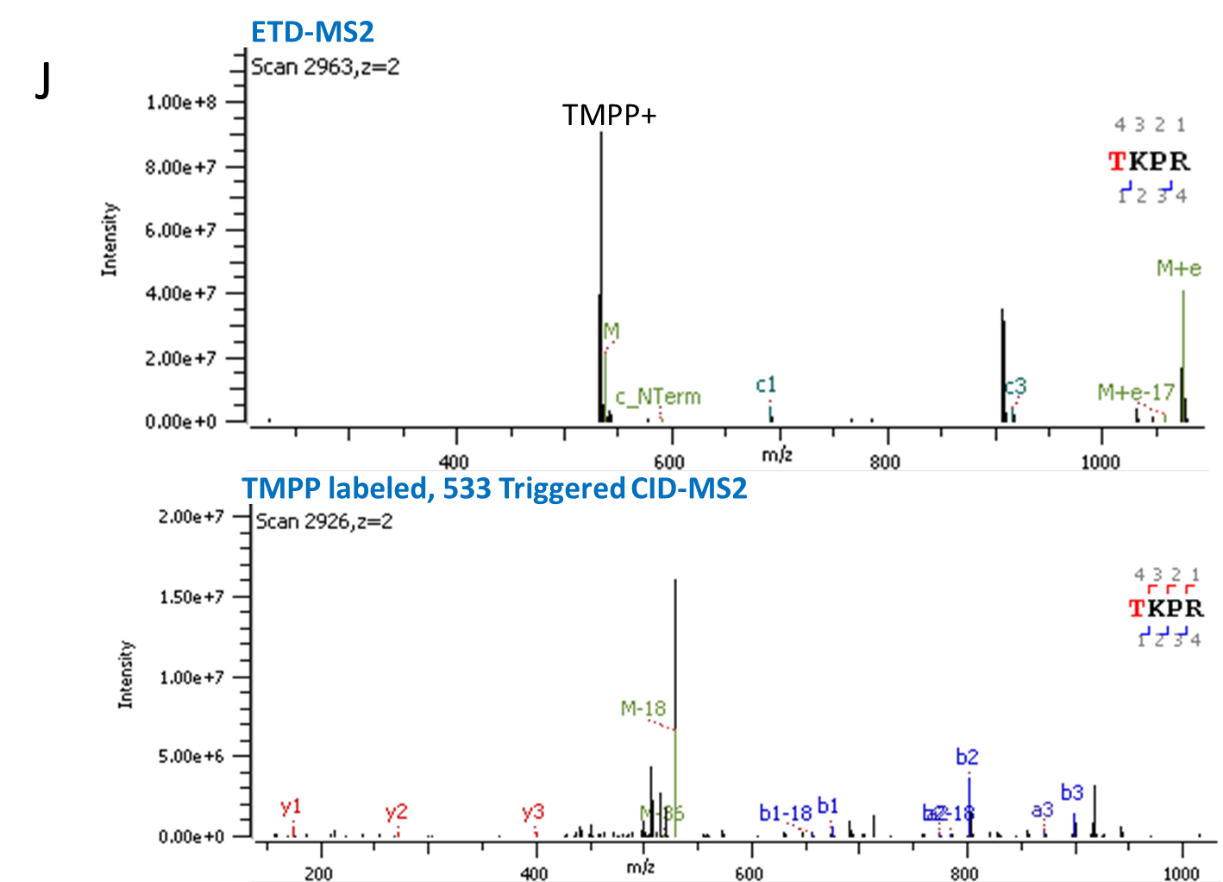


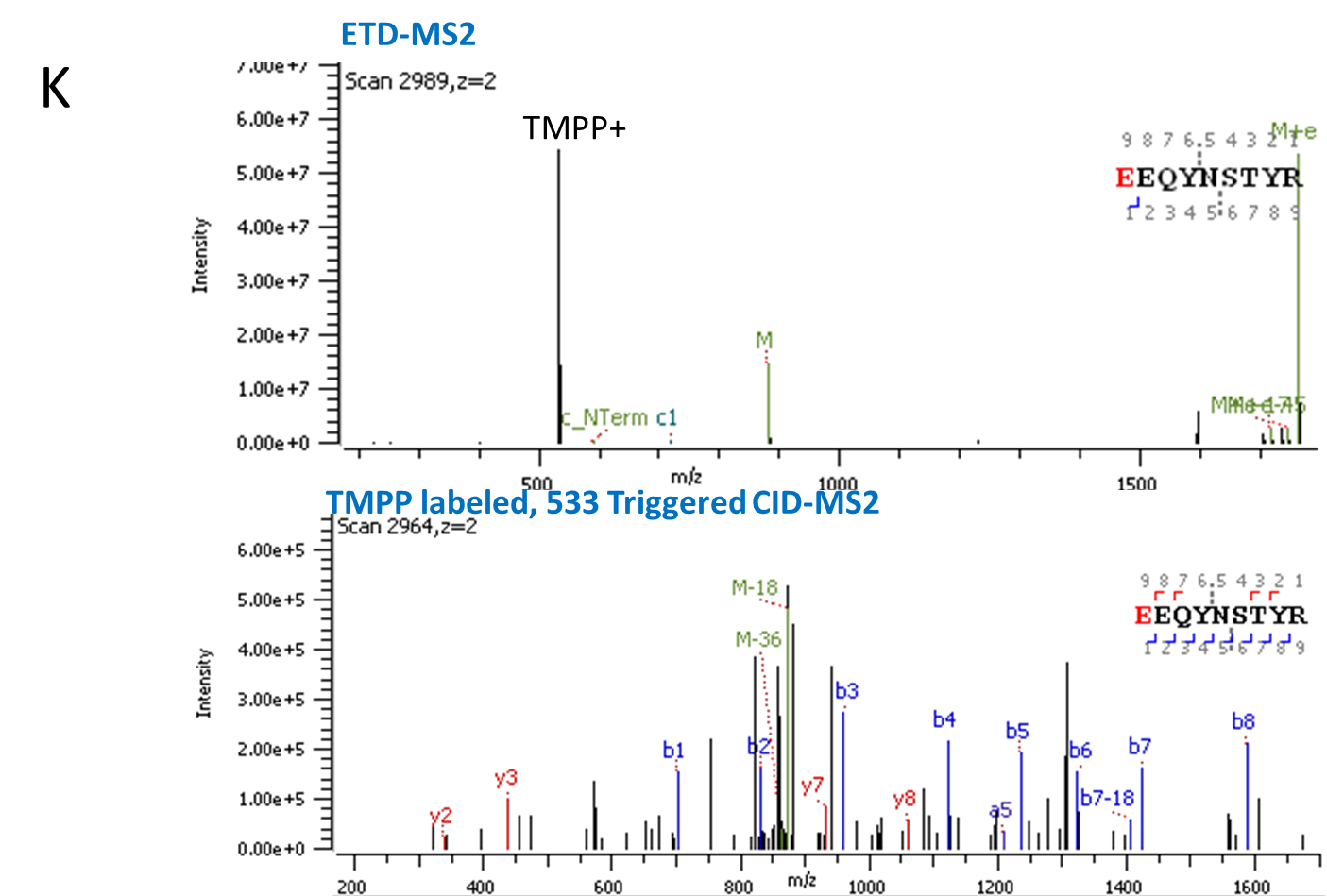


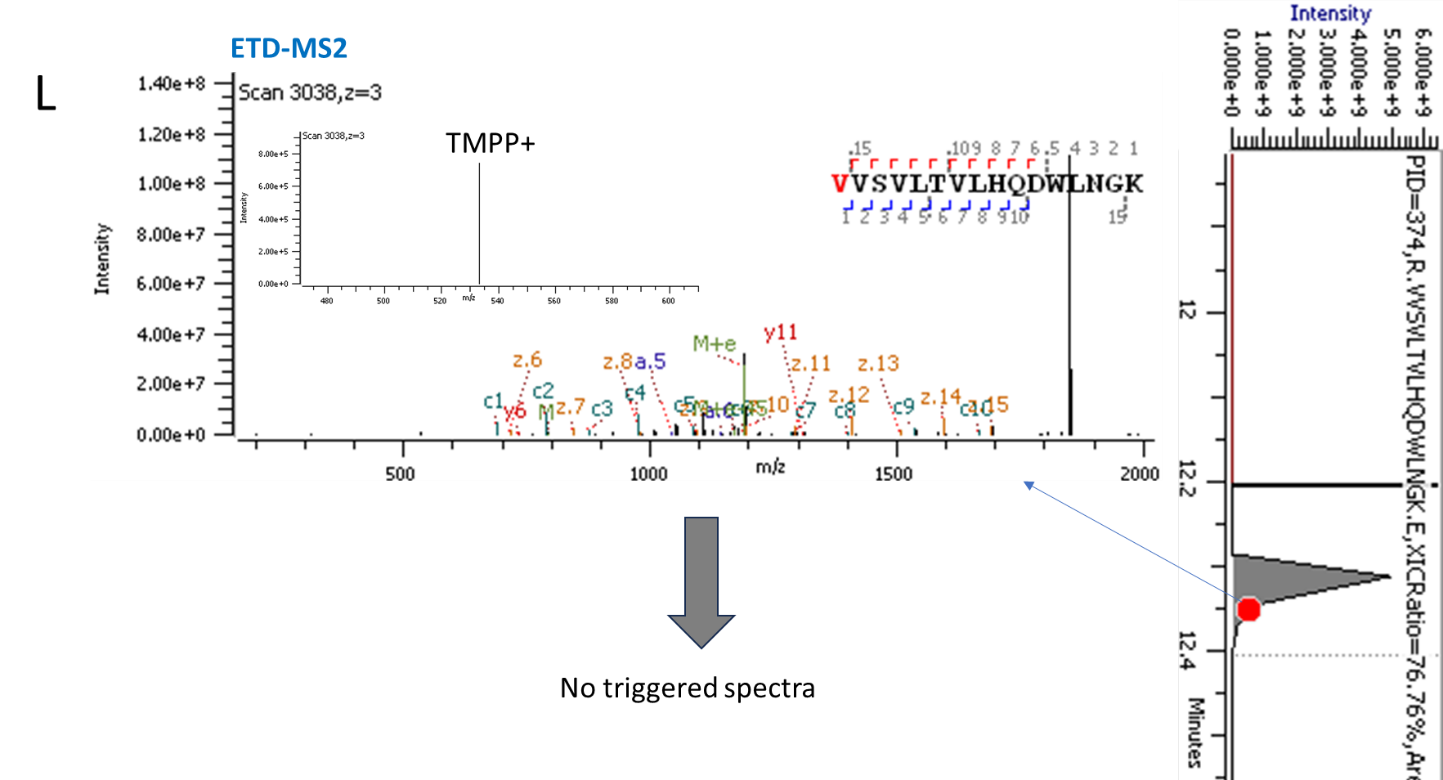


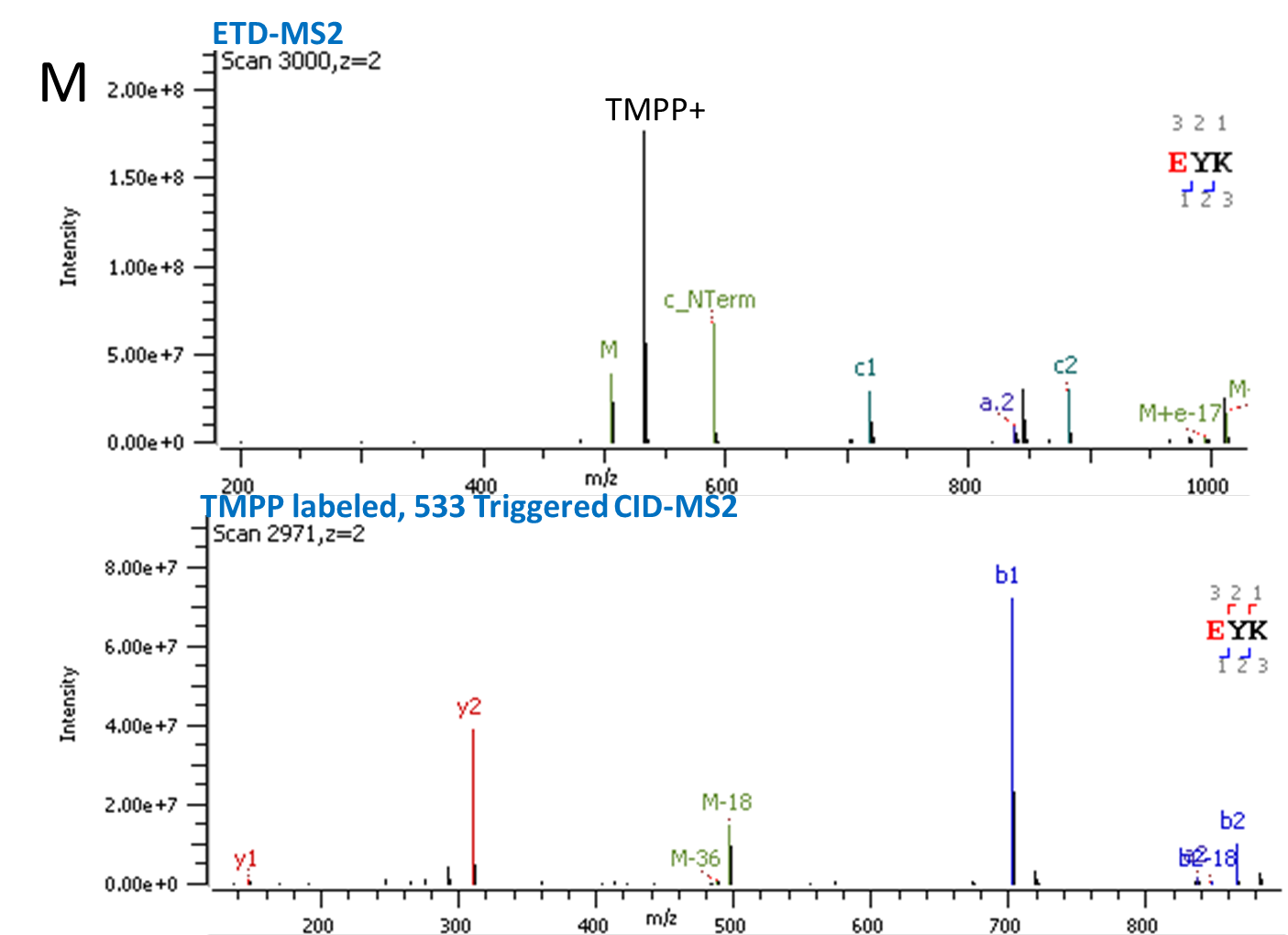


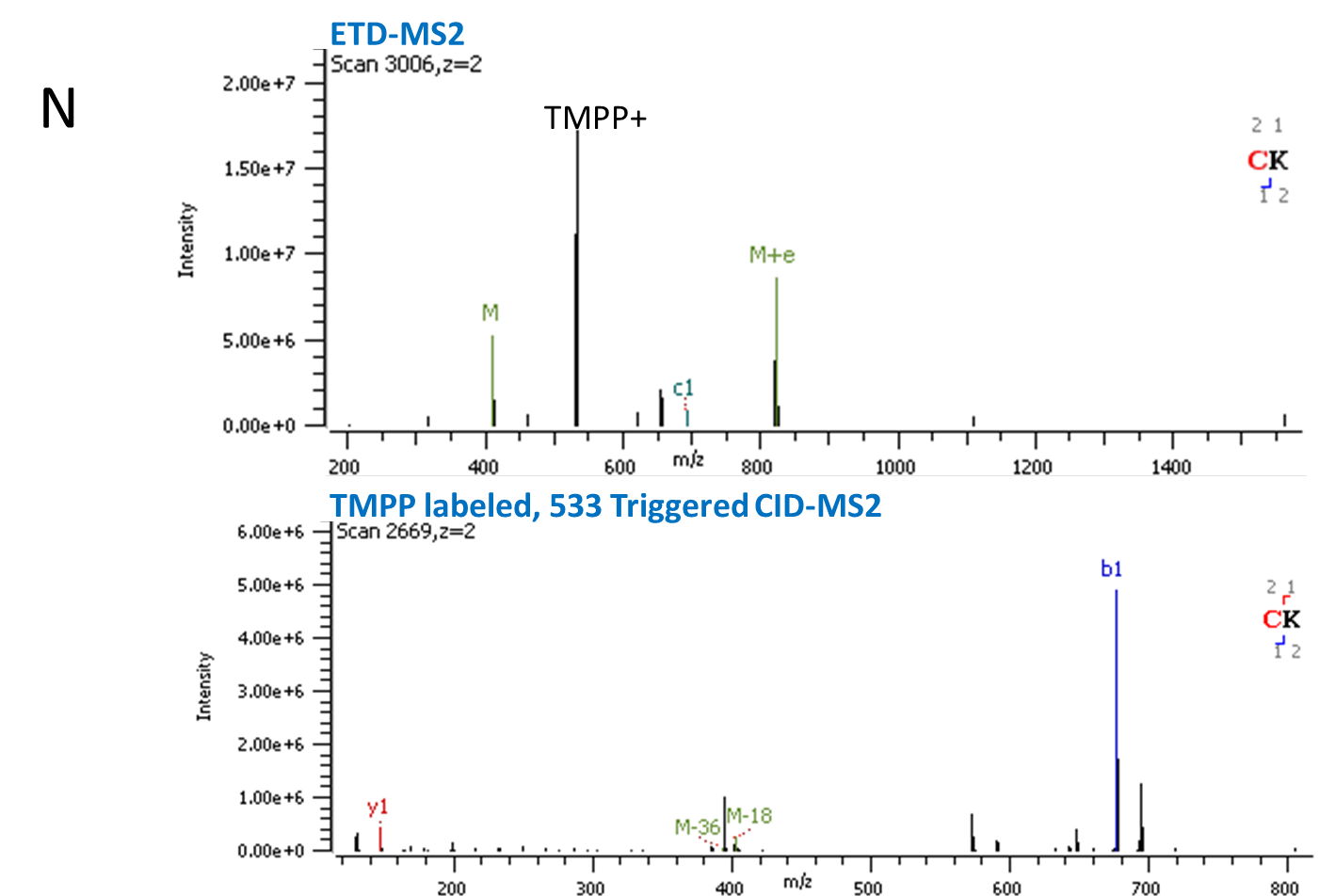


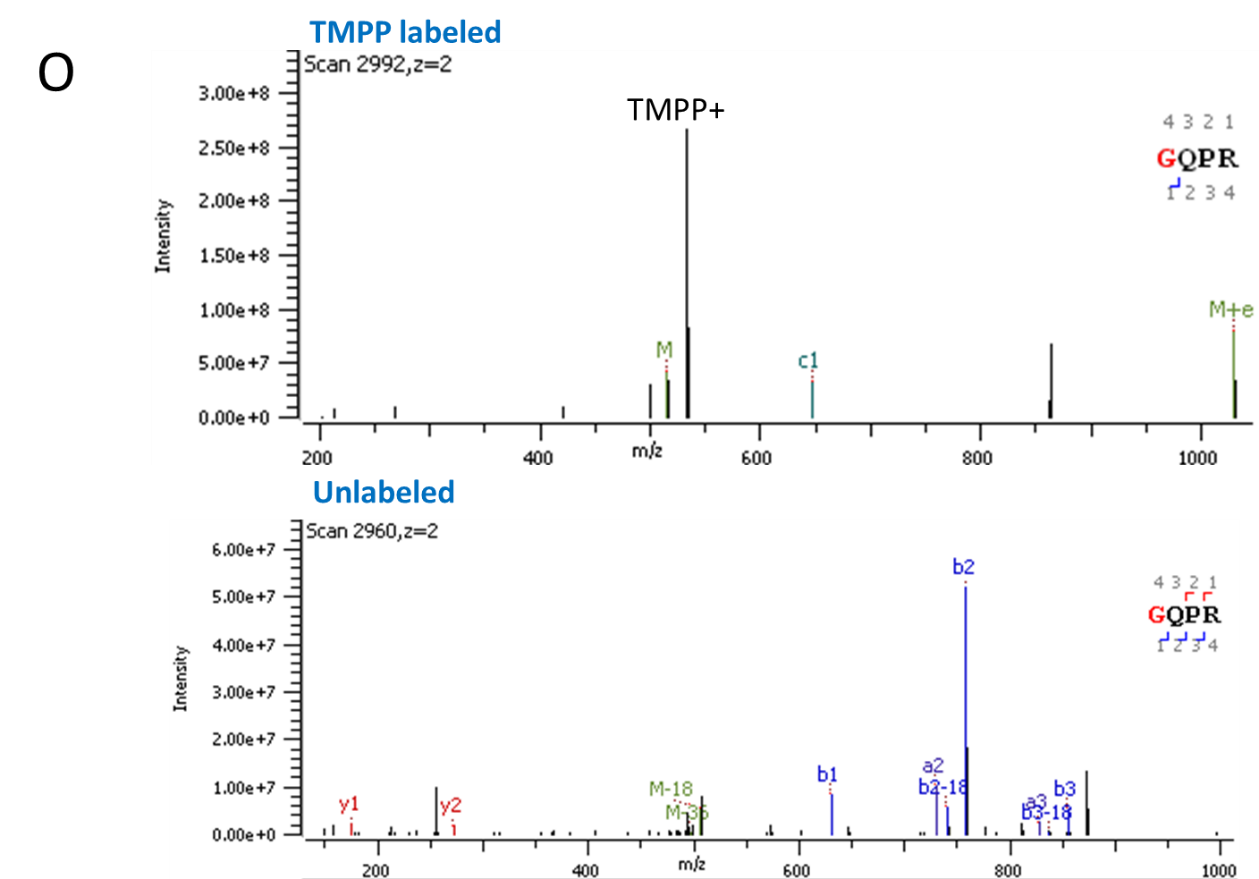


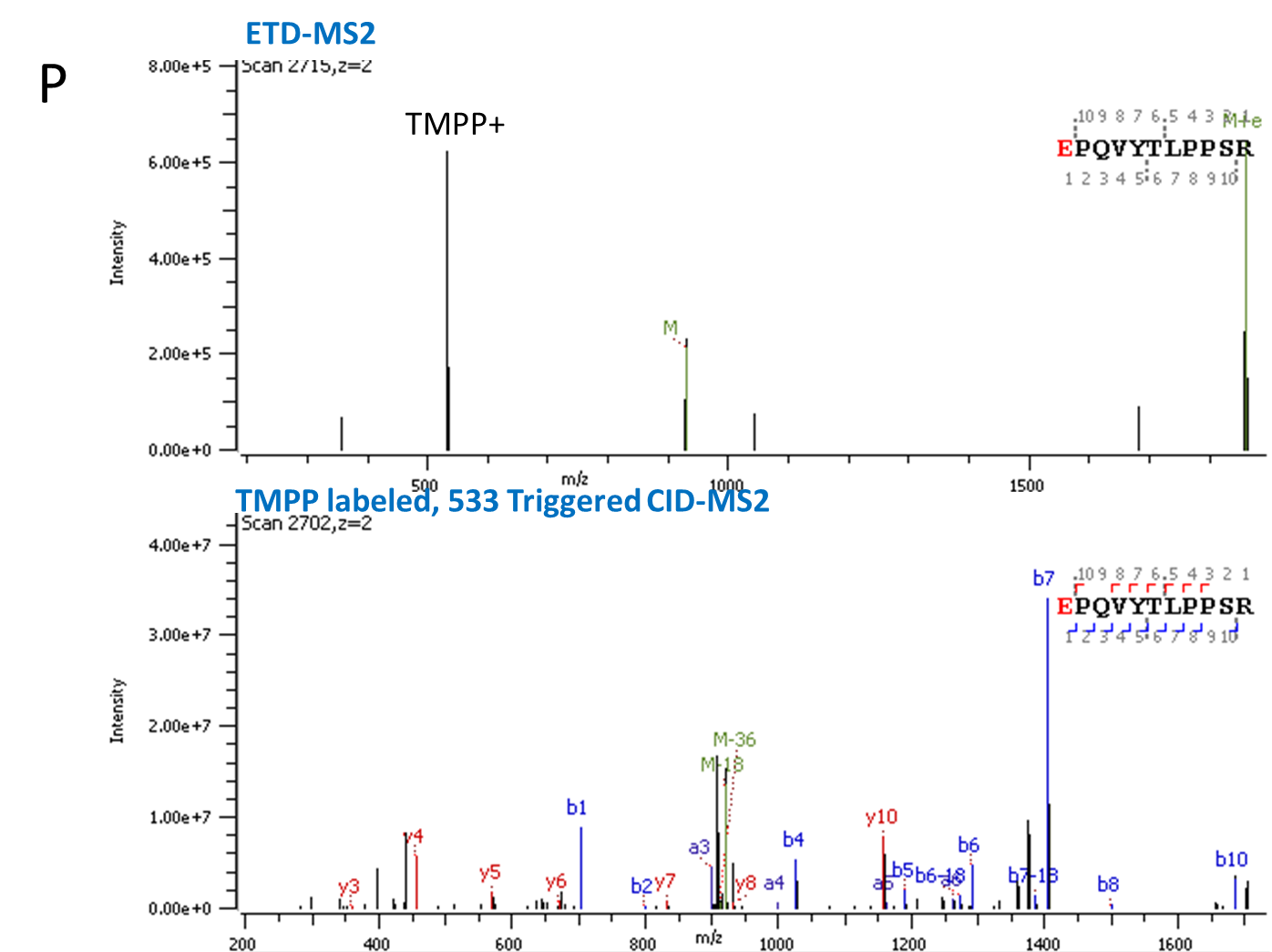


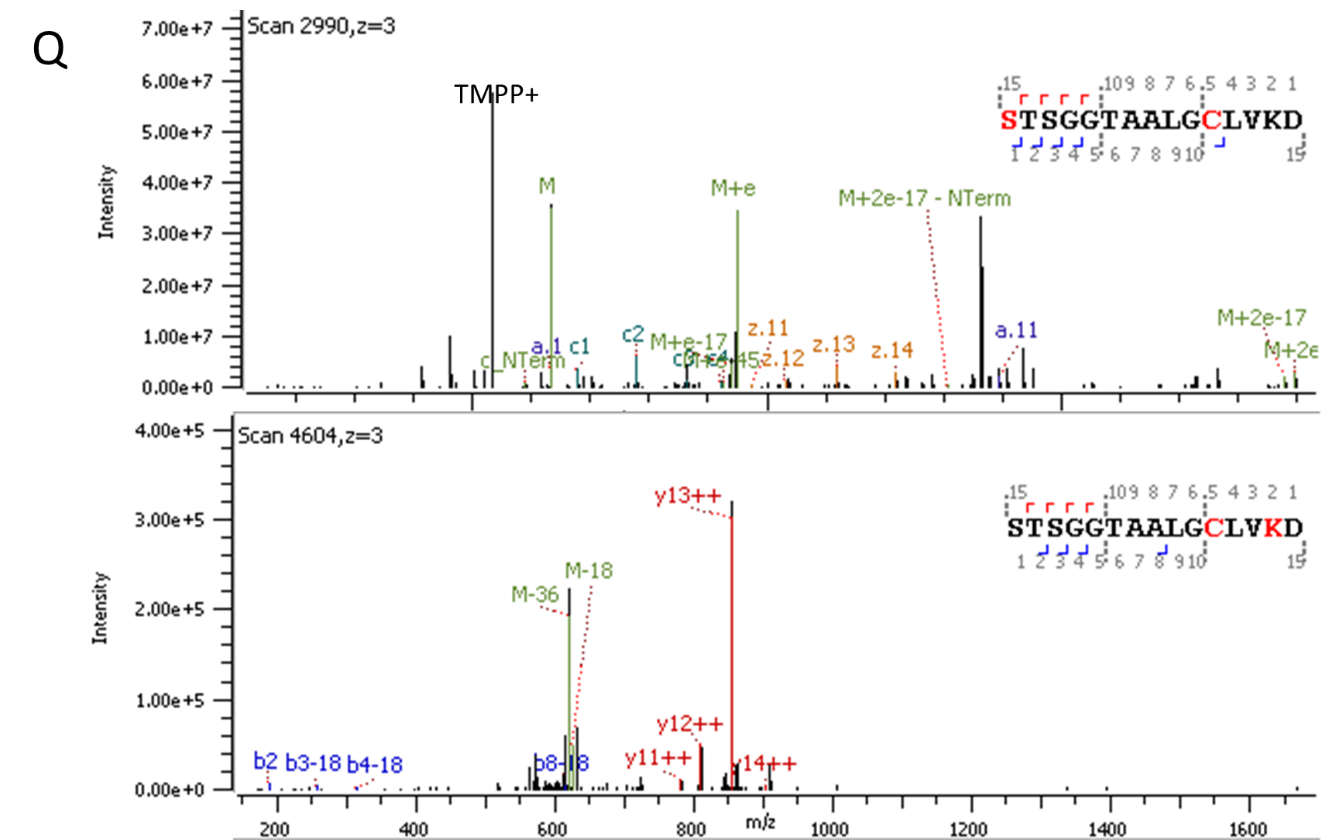


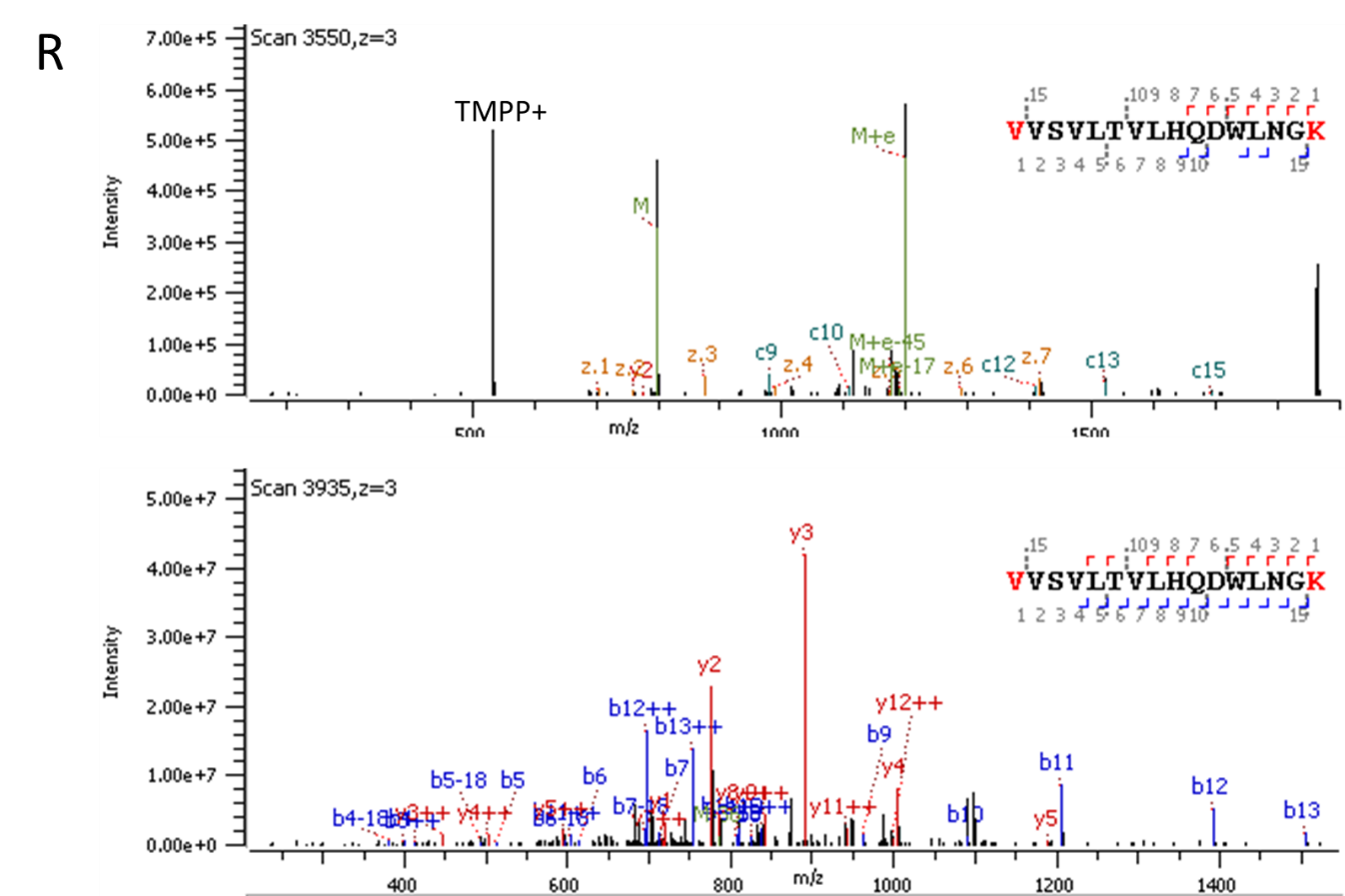


Fig S2A-R. NIST mAb synthetic peptide standards with TMPP subjected to LC-MS using ETD-MS2 and diagnostic reporter (TMPP+, (533 Da) ion triggered-MS2-CID events of peptides with different lengths, amino acid composition and labeling sites.


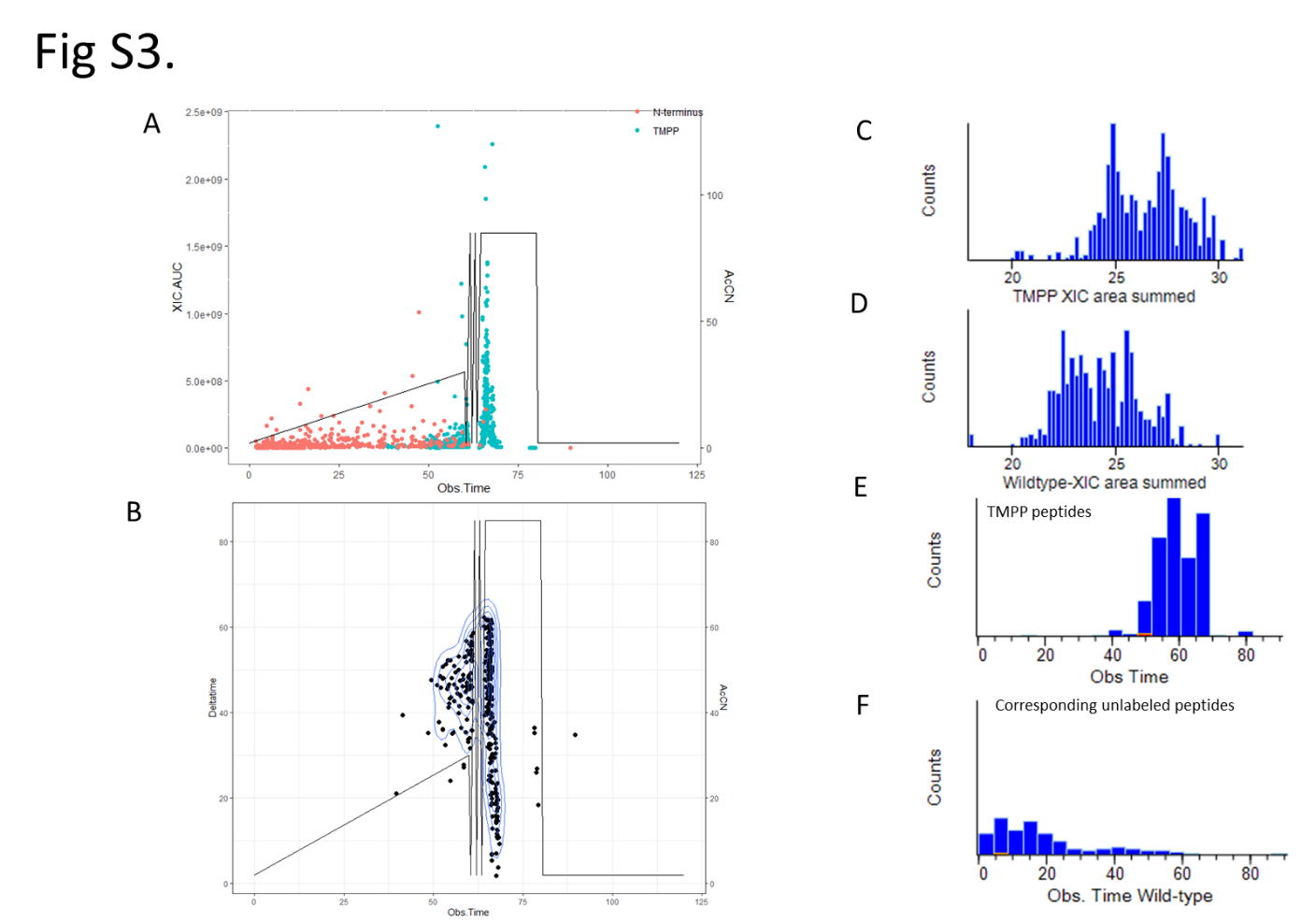


Fig S3. LC-MS of K562 derived tryptic peptides labeled with TMPP reagents. (A) Peptide XIC intensity as function of retention time overlaid with AcCN gradient. (B) TMPP labeled peptide density as a function of retention time overlaid with AcCN gradient. (C) TMMP labeled peptide XIC area counts (D) Unlabeled wild-type peptide XIC area counts (E) Retention time distribution of TMPP labeled peptides (F) Retention time distribution of unlabeled wild-type peptides.


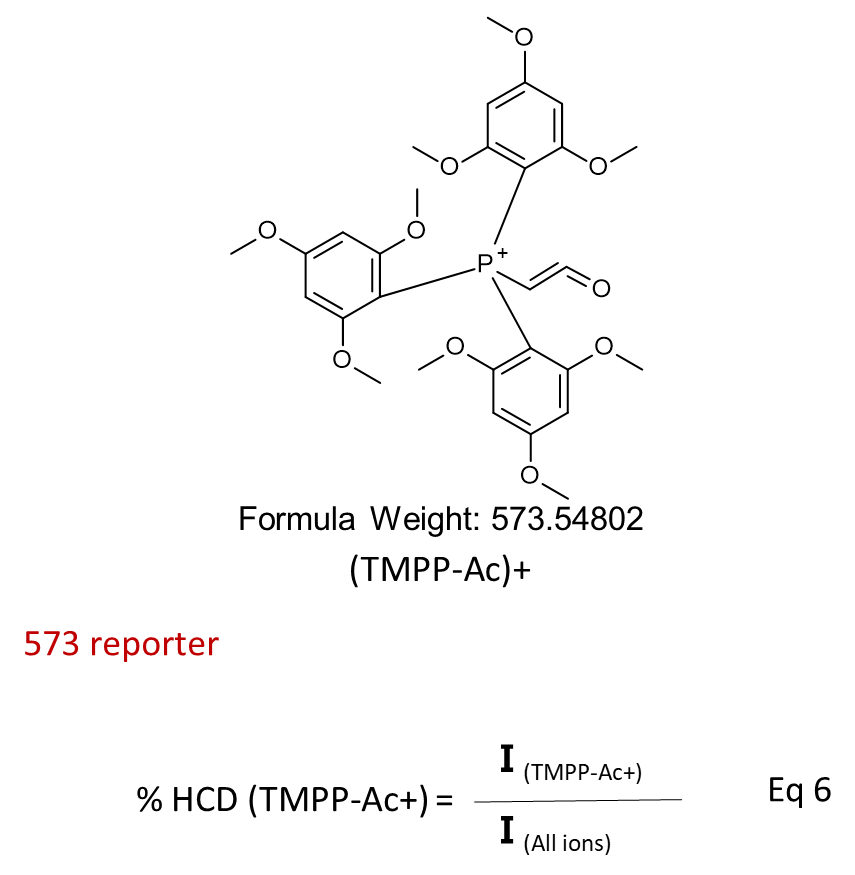

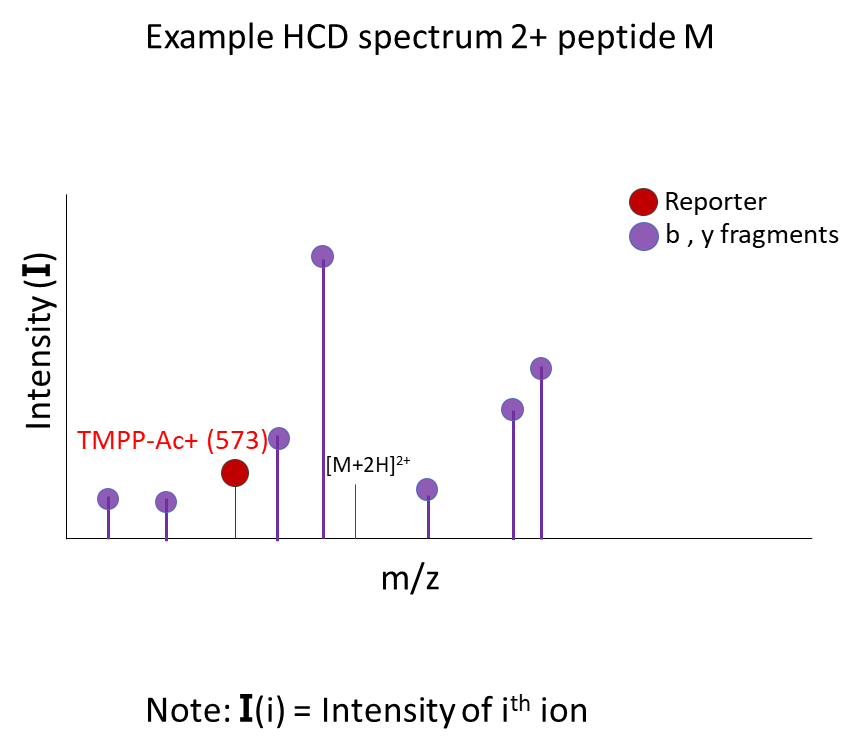


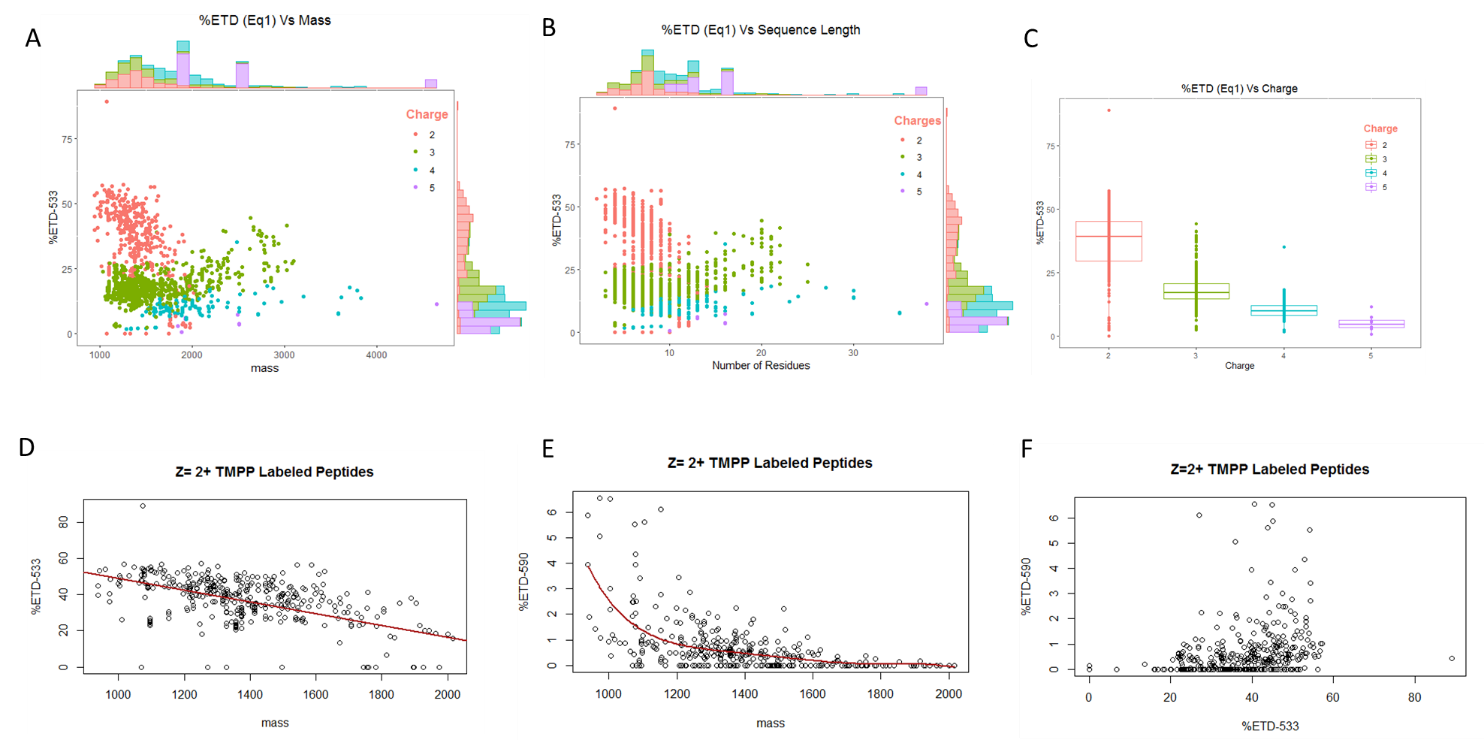
Fig S4. High-energy Collision Dissociation (HCD) efficiency estimated using TMPP derived reporter ions: (TMPP-Ac+ (573 Da)) and peptide backbone b- and y-type fragment ions. Eq6 are %HCD efficiency estimated using Intensities (I) of the reporter ion and backbone fragment ions.

Fig S5. Electron transfer dissociation (ETD) efficiency estimated using TMPP derived reporter ions: TMPP+ (533 Da), TMPP-Ac-NH2+ (590 Da) and peptide backbone c- and z-type fragment ions as in Eq1. (A) %ETD-533 as a function of peptide mass with peptides grouped by charge state (B) %ETD-590 as a function of peptide length with peptides grouped by charge state (C) %ETD-533 as a function of peptides charge state (D) %ETD-533 as a function of mass of TMPP labeled (+2) peptides. (E) %ETD-590 as a function of mass of TMPP labeled (+2) peptides. (F) %ETD-590 versus %ETD-533 for TMPP labeled (2+) peptides


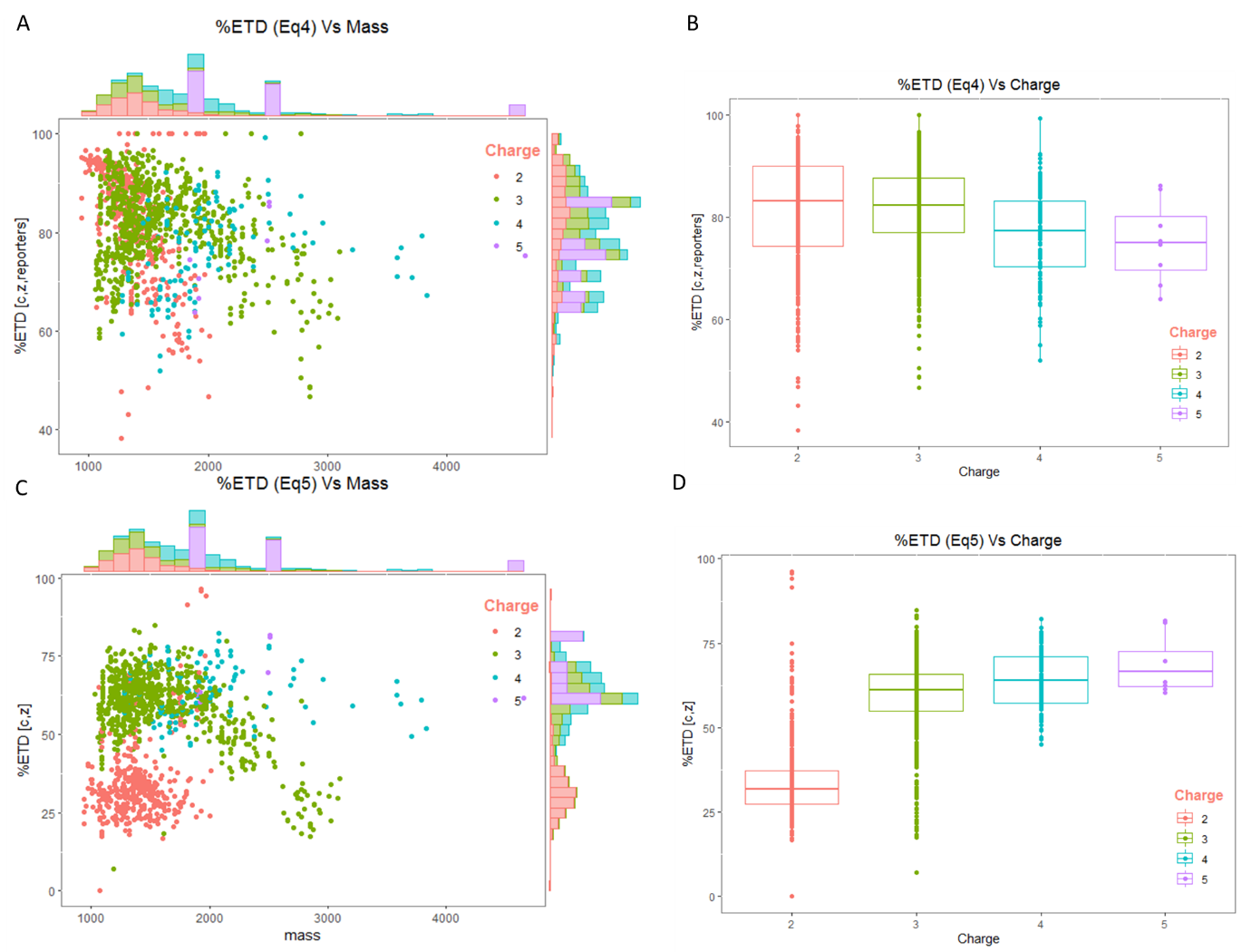


Fig S6. Electron transfer dissociation (ETD) efficiency estimated using TMPP derived reporter ions: TMPP+ (533 Da), TMPP-Ac-NH2+ (590 Da) and peptide backbone c- and z-type fragment ions, (A) %ETD, using reporters and c, z ions as a function of mass (Eq4) (B) %ETD, using reporters and c, z ions as a function of charge state (Eq4) (C) %ETD, using c, z ions as a function of mass (Eq5) (D) %ETD, using c, z ions as a function of charge state (Eq5)


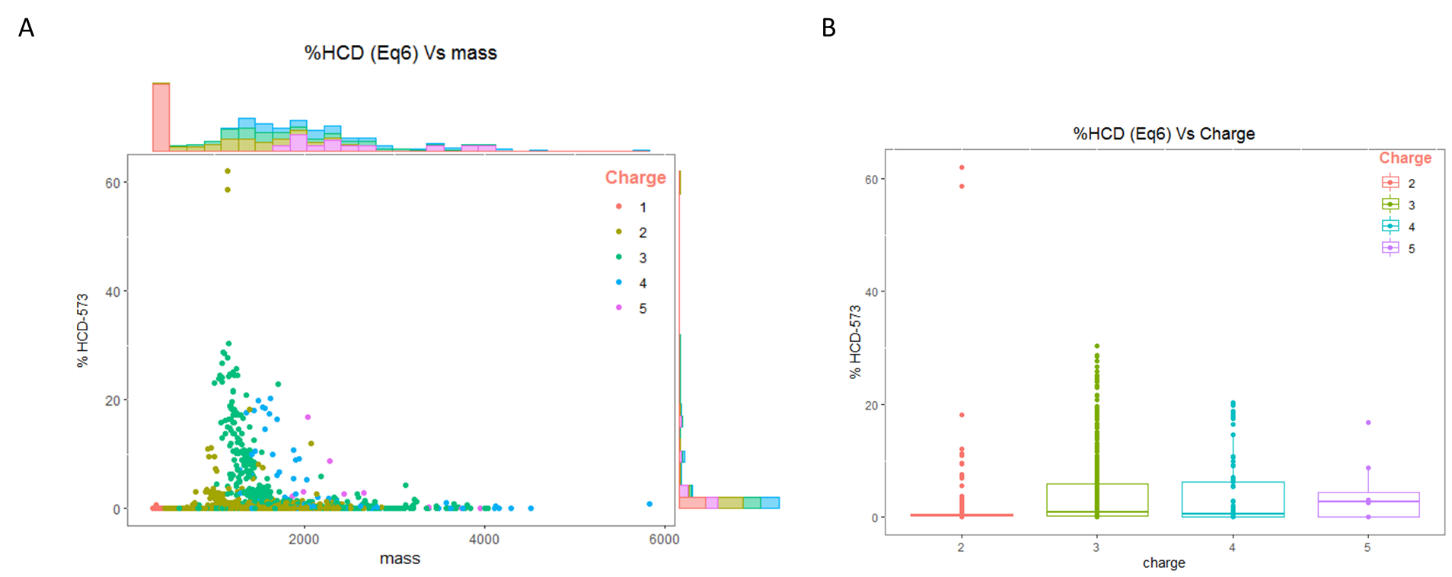


Fig S7. High-energy Collisional Dissociation (HCD) efficiency estimated using TMPP derived reporter ions: TMPP-Ac+ (573 Da), and peptide backbone b- and y-type fragment ions, (A) %HCD -573 Da reporter as a function of mass of peptides with peptides grouped by charge state (Eq6) (B) %HCD-573 Da reporter as a function of peptide charge state (Eq6)


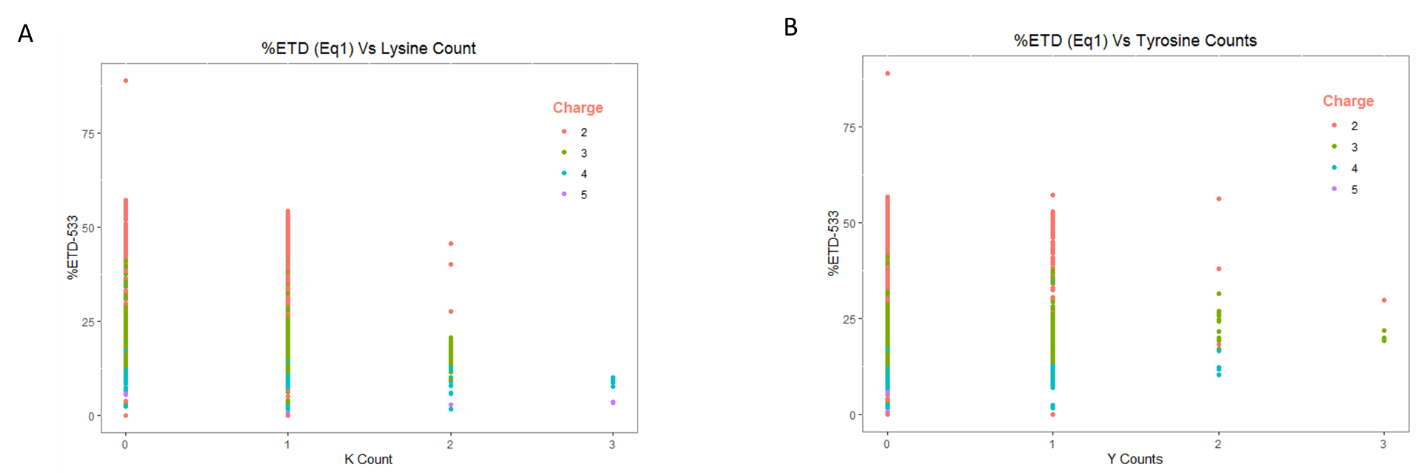


Fig S8. ETD efficiency estimated using (TMPP-Ac+ (533 Da)) ions using Eq1, (A) %ETD as a function of the TMMP labeled at Lysine (K) residues or K count. (B) %ETD as a function of the TMMP labeled at Tyrosine (Y) residues or Y count.


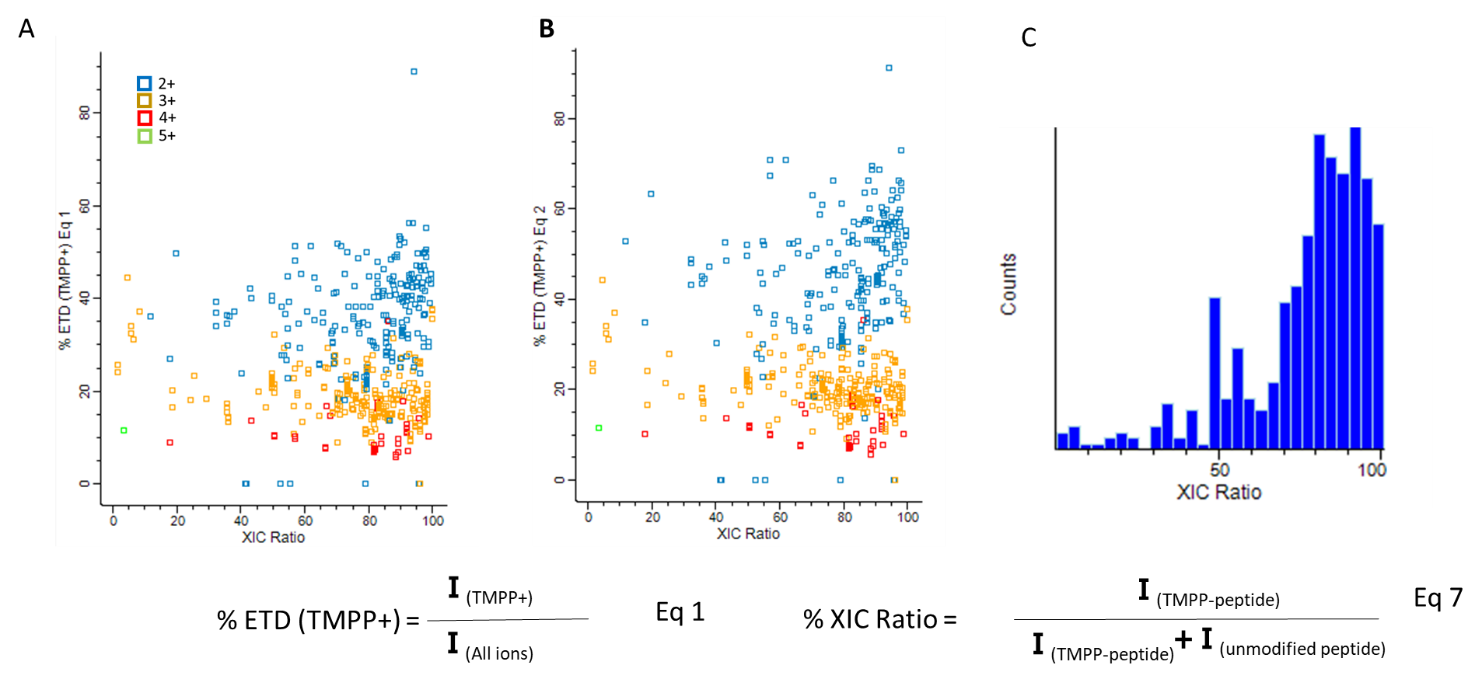


Fig S9. %ETD as a function of XIC ratio or %TMPP labeled peptides (A) %ETD estimated using Eq 1. and peptides grouped by charge state. (B) %ETD estimated using Eq 2. and peptides grouped by charge state (C) The total peptide counts as a function of %TMPP labeled peptides


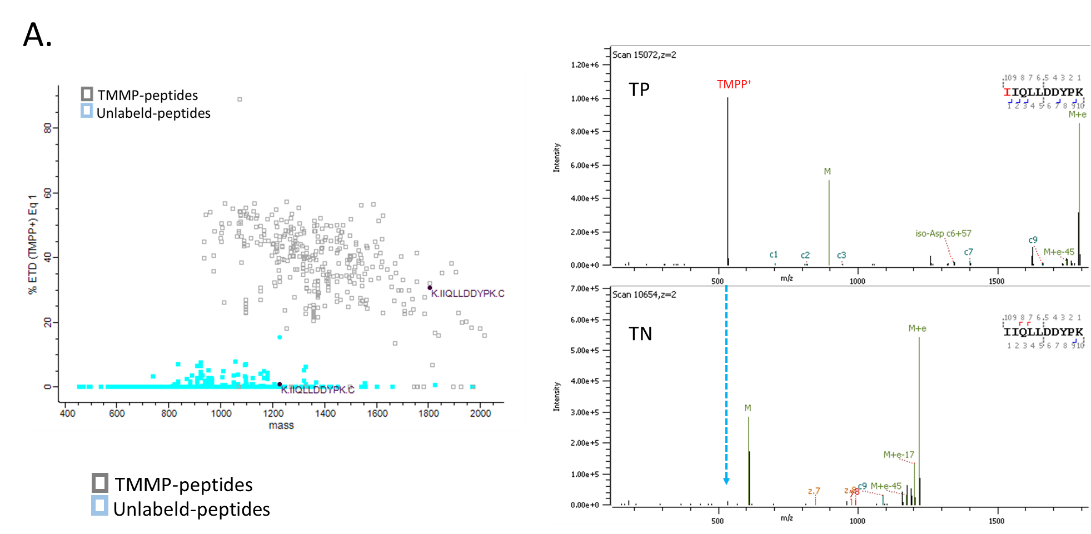

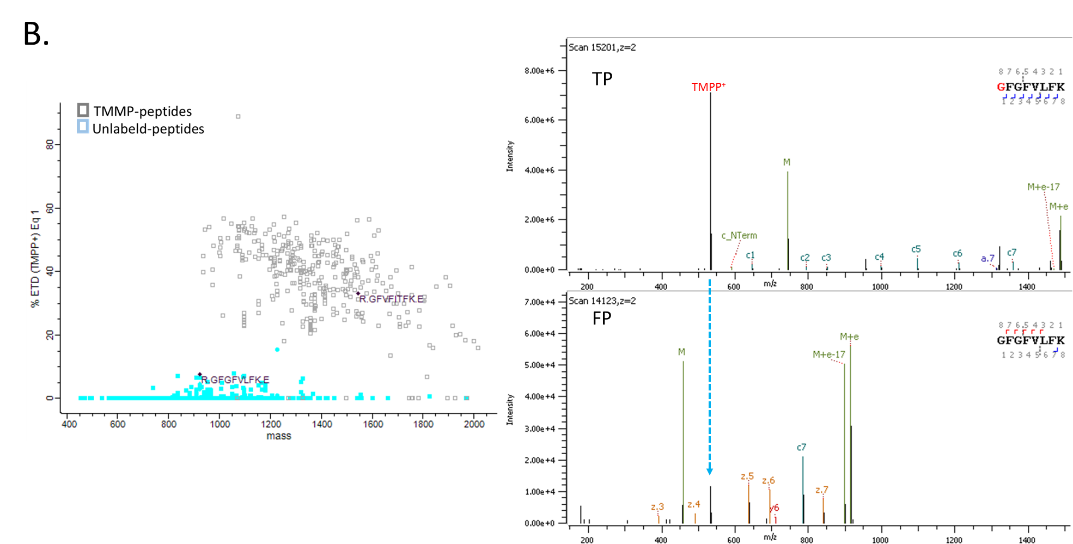

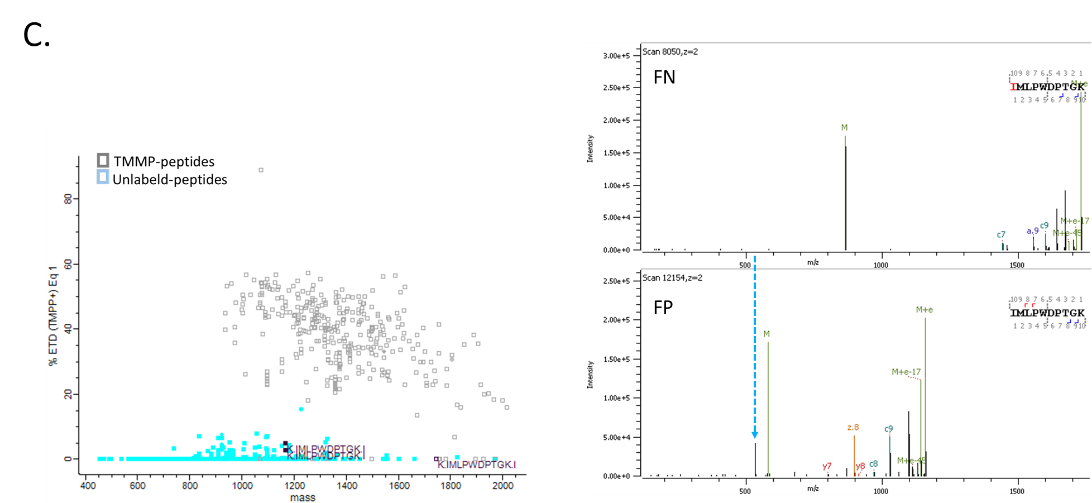


Fig S10. Representative ETD-MS2 spectra of TMPP labeled and unlabeled pairs for given sequence where TMPP+ (533) reporter ion abundance is used as a feature to classify spectra that are diagnostic of a clipped site (A) True Positive (TP) TMPP labeled peptide with a True Negative unlabeled peptide counterpart (B) True Positive (TP) TMPP labeled peptide with False Positive unlabeled peptide counterpart, Note: The 533 diagnostic ion in the spectrum was confirmed was due to the co-solation and co-fragmentation of 3+ TMPP labeled TPEEGEK interference peptide (C) False Negative (FN) TMPP labeled peptide with False Positive unlabeled peptide counterpart.


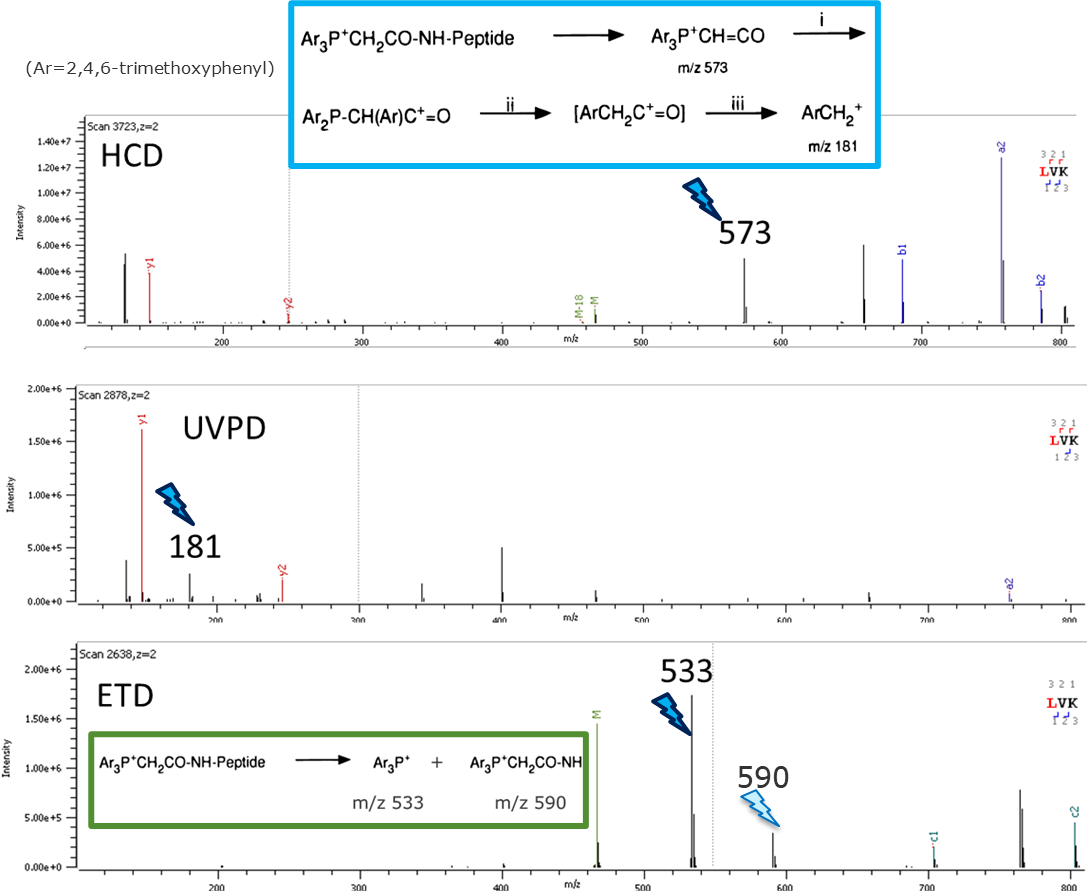


Fig S11. Diagnostic ions generated for TMPP labeled LVK peptide in GLP1-Fc fusion protein (A) HCD-MS2 reporter: Ar_3_P-Ac+ (573 Da), (B) UVPD-MS2 reporter: Ar-CH_2_+ (181 Da) (C) ETD-MS2 reporters: Ar_3_P+ (533) and Ar_3_PCH_2_CONH_2_+ (590). Note: The scheme for diagnostic ion at 181 Da was due to further dissociation of 573 Da reporter ion^1^.

**Table S1.** Dissociation Methods and Identified Clip sites of GLP1 based on TMPP-specific diagnostic ions and sequence ions.


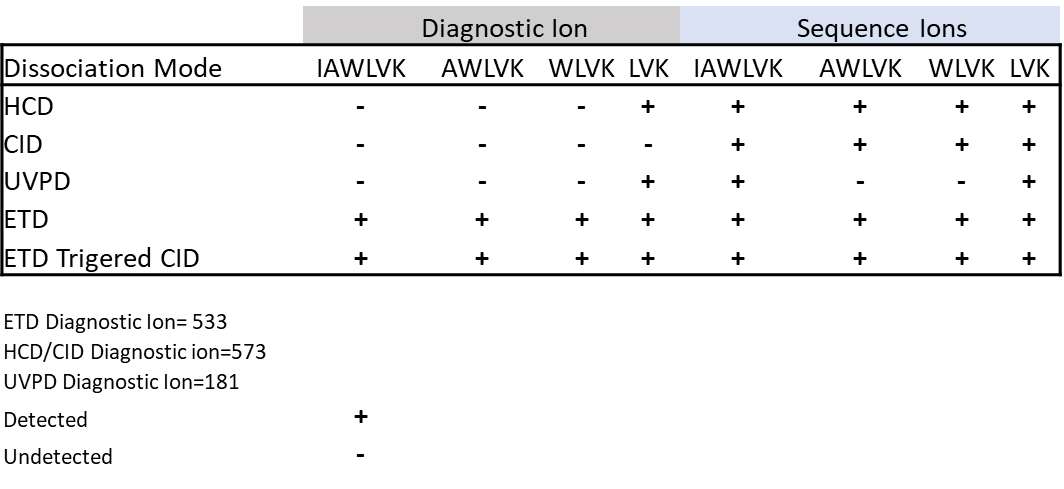


**REFERENCES**

1. Huang, Z.H. et al. A picomole-scale method for charge derivatization of peptides for sequence analysis by mass spectrometry. *Analytical chemistry* **69**, 137-144 (1997).
